# Identifying Mixtures of Mixtures Using Bayesian Estimation

**DOI:** 10.1080/10618600.2016.1200472

**Published:** 2017-04-24

**Authors:** Gertraud Malsiner-Walli, Sylvia Frühwirth-Schnatter, Bettina Grün

**Affiliations:** ^a^ Department of Applied Statistics, Johannes Kepler University, Linz, Austria; ^b^ Institute of Statistics and Mathematics, Wirtschaftsuniversität, Wien, Austria

**Keywords:** Bayesian nonparametric mixture model, Dirichlet prior, Finite mixture model, Model-based clustering, Normal gamma prior, Number of components

## Abstract

The use of a finite mixture of normal distributions in model-based clustering allows us to capture non-Gaussian data clusters. However, identifying the clusters from the normal components is challenging and in general either achieved by imposing constraints on the model or by using post-processing procedures. Within the Bayesian framework, we propose a different approach based on sparse finite mixtures to achieve identifiability. We specify a hierarchical prior, where the hyperparameters are carefully selected such that they are reflective of the cluster structure aimed at. In addition, this prior allows us to estimate the model using standard MCMC sampling methods. In combination with a post-processing approach which resolves the label switching issue and results in an identified model, our approach allows us to simultaneously (1) determine the number of clusters, (2) flexibly approximate the cluster distributions in a semiparametric way using finite mixtures of normals and (3) identify cluster-specific parameters and classify observations. The proposed approach is illustrated in two simulation studies and on benchmark datasets. Supplementary materials for this article are available online.

## 1. Introduction

In many areas of applied statistics like economics, finance, or public health it is often desirable to find groups of similar objects in a dataset through the use of clustering techniques. A flexible approach to clustering data is based on mixture models, whereby the data in each mixture component are assumed to follow a parametric distribution with component-specific parameters varying over the components. This so-called model-based clustering approach (Fraley and Raftery 2002) is based on the notion that the component densities can be regarded as the “prototype shape of clusters to look for” (Hennig 2010) and each mixture component may be interpreted as a distinct data cluster.

Most commonly, a finite mixture model with Gaussian component densities is fitted to the data to identify homogeneous data clusters within a heterogeneous population. However, assuming such a simple parametric form for the component densities implies a strong assumption about the shape of the clusters and may lead to overfitting the number of clusters as well as a poor classification, if not supported by the data. Hence, a major limitation of Gaussian mixtures in the context of model-based clustering results from the presence of non-Gaussian data clusters, as typically encountered in practical applications.

Recent research demonstrates the usefulness of mixtures of parametric non-Gaussian component densities such as the skew normal or skew-*t* distribution to capture non-Gaussian data clusters, see Frühwirth-Schnatter and Pyne (2010), Lee and McLachlan (2014), and Vrbik and McNicholas (2014), among others. However, as stated by Li (2005), for many applications it is difficult to decide which parametric distribution is appropriate to characterize a data cluster, especially in higher dimensions. In addition, the shape of the cluster densities can be of a form which is not easily captured by a parametric distribution. To better accommodate such data, recent advances in model-based clustering focused on designing mixture models with more flexible, not necessarily parametric cluster densities.

A rather appealing approach, known as mixture of mixtures, models the non-Gaussian cluster distributions themselves by Gaussian mixtures, exploiting the ability of normal mixtures to accurately approximate a wide class of probability distributions. Compared to a mixture with Gaussian components, mixture of mixtures models impose a two-level hierarchical structure which is particularly appealing in a clustering context. On the higher level, Gaussian components are grouped together to form non-Gaussian cluster distributions which are used for clustering the data. The individual Gaussian component densities appearing on the lower level of the model influence the clustering procedure only indirectly by accommodating possibly non-Gaussian, but otherwise homogeneous cluster distributions in a semiparametric way. This powerful and very flexible approach has been employed in various ways, both within the framework of finite and infinite mixtures.

Statistical inference for finite mixtures is generally not easy due to problems such as label switching, spurious modes and unboundedness of the mixture likelihood (see, e.g., Frühwirth-Schnatter 2006, chap. 2), but estimation of a mixture of mixtures model is particularly challenging due to additional identifiability issues. Since exchanging subcomponents between clusters on the lower level leads to different cluster distributions, while the density of the higher level mixture distribution remains the same, a mixture of mixtures model is not identifiable from the mixture likelihood in the absence of additional information. For example, strong identifiability constraints on the locations and the covariance matrices of the Gaussian components were imposed by Bartolucci (2005) for univariate data and by Di Zio, Guarnera, and Rocci (2007) for multivariate data to estimate identified finite mixtures of Gaussian mixtures.

A different strand of literature pursues the idea of creating meaningful clusters after having fitted a standard Gaussian mixture model to the data. The clusters are determined by successively merging components according to some criterion, for example, the closeness of the means (Li 2005), the modality of the obtained mixture density (Chan et al. 2008; Hennig 2010), the degree of overlapping measured by misclassification probabilities (Melnykov 2016), or the entropy of the resulting partition (Baudry et al. 2010). However, such two-step approaches might miss the general cluster structure, see Appendix E for an example.

In the present article, we identify the mixture of mixtures model within a Bayesian framework through a hierarchical prior construction and propose a method to simultaneously select a suitable number of clusters. In our approach, both the identification of the model and the estimation of the number of clusters is achieved by employing a selectively informative prior on the model parameters.

Our choice of prior parameters is driven by assumptions on the cluster shapes assumed to be present in the data, thus being in line with Hennig (2010) who emphasizes that, *“it rather has to be decided by the statistician under which conditions different Gaussian mixture components should be regarded as a common cluster.”* This prior specification introduces dependence among the subcomponent densities within each cluster, by pulling the subcomponent means on the lower level toward the cluster center, making the cluster distributions themselves dense and connected. On the higher level, the prior is based on the notion that the cluster centers are quite distinct from each other compared to the spread of the clusters. The choice of the hyperparameters of this hierarchical prior turns out to be crucial in achieving identification and is guided by a variance decomposition of the data.

Regarding the estimation of the number of clusters, a sparse hierarchical mixture of mixtures model is derived as an extension of the sparse finite mixture model introduced by Malsiner-Walli, Frühwirth-Schnatter, and Grün (2016). There, based on theoretical results derived by Rousseau and Mengersen (2011), an overfitting Gaussian mixture with *K* components is specified where a sparse prior on the mixture weights has the effect of assigning the observations to fewer than *K* components. Thus, the number of clusters can be estimated by the most frequent number of nonempty components encountered during Markov chain Monte Carlo (MCMC) sampling. In this article, rather than using a single multivariate Gaussian distribution, we model the component densities in a semiparametric way through a Gaussian mixture distribution, and again use a sparse prior on the cluster weights to automatically select a suitable number of clusters on the upper level.

Specifying a sparse prior on the weights is closely related to Bayesian nonparametric (BNP) Gaussian mixture models such as Dirichlet process mixtures (DPMs; Ferguson 1983; Escobar and West 1995). The sparse prior on the cluster weights induces clustering of the observations, similar as for DPMs which have been applied in a clustering context by Quintana and Iglesias (2003), Medvedovic, Yeung, and Bumgarner (2004), and Dahl (2006), among others. The hierarchical mixture of mixtures model we introduce is similar to hierarchical BNP approaches such as the hierarchical DPM (Teh et al. 2006). Very closely related BNP approaches are the nested DPM (Rodriguez, Dunson, and Gelfand 2008), the infinite mixture of infinite Gaussian mixtures (Yerebakan, Rajwa, and Dundar 2014), and species mixture models (Argiento, Cremaschi, and Guglielmi 2014) which directly work on the partition of the data. We discuss in Sections 2.4 and 3.1 similarities as well as differences between our approach and BNP models.

We finally note that the implementation effort to estimate our model is moderate and standard MCMC methods based on data augmentation and Gibbs sampling (see Frühwirth-Schnatter 2006) can be used. Several approaches proposed in the literature can be used to post-process the MCMC draws to obtain a clustering of the data and to perform cluster-specific inference. For our simulation studies and applications, we adapt and extend the method suggested by Frühwirth-Schnatter (2006, 2011) which determines a unique labeling for the MCMC draws by clustering the draws in the point process representation.

The rest of the article is organized as follows. Section 2 describes the proposed strategy, including detailed prior specifications, and relates our method to the two-layer BNP approaches in Rodriguez, Dunson, and Gelfand (2008) and Yerebakan, Rajwa, and Dundar (2014). Clustering and model estimation issues are discussed in Section 3. The performance of the proposed strategy is evaluated in Section 4 in simulation studies and for various benchmark datasets. Section 5 concludes.

## 2. Sparse Hierarchical Mixture of Mixtures Model

### 2.1 Model Definition

Following previous work on hierarchical mixtures of mixtures, we assume that *N* observations **y**_*i*_, *i* = 1, …, *N* of dimension 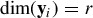 are drawn independently from a finite mixture distribution with *K* components,
(1)
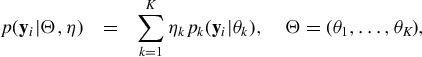
with each component distribution 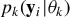 being a mixture of *L* normal subcomponents:
(2)
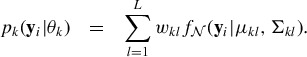
To distinguish the component distributions on the upper level from the Gaussian components on the lower level, we will refer to the former ones as “cluster distributions”. For clustering the observations based on Bayes’ rule, the cluster weights 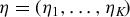 and the cluster densities 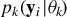 on the upper level (1) are relevant.

Since the number of data clusters is unknown and needs to be inferred from the data, we assume that (1) is an overfitting mixture, that is, the specified number of clusters *K* exceeds the number of clusters present in the data. Following the concept of sparse finite mixtures (Malsiner-Walli, Frühwirth-Schnatter, and Grün 2016), we choose a symmetric Dirichlet distribution as prior for the weight distribution, that is, 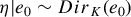, and base our choice of *e*_0_ on the results of Rousseau and Mengersen (2011) concerning the asymptotic behavior of the posterior distribution of an overfitting mixture model. They show that this behavior is determined by the hyperparameter *e*_0_ of the Dirichlet prior on the weights. In particular, they prove that, if *e*_0_ < *d*/2, where *d* is the dimension of the cluster-specific parameters 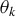, then the posterior expectation of the weights associated with superfluous clusters asymptotically converges to zero.

Hence, we specify a sparse prior on the cluster weights 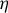 by choosing *e*_0_ ≪ *d*/2 so that superfluous clusters are emptied during MCMC sampling and the number of nonempty clusters on the cluster level is an estimator for the unknown number of data clusters. In this way, the specification of a sparse cluster weight prior in an overfitting mixture of mixtures model provides an “automatic tool” to select the number of clusters, avoiding the expensive computation of marginal likelihoods as, for example, in Frühwirth-Schnatter (2004). Empirical results in Malsiner-Walli, Frühwirth-Schnatter, and Grün (2016) indicate that *e*_0_ needs to be chosen very small, for example, *e*_0_ = 0.001, to actually empty all superfluous clusters in the finite sample case.

On the lower level (2), in each cluster *k*, a semiparametric approximation of the cluster distributions is achieved by mixing *L* multivariate Gaussian subcomponent densities 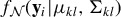, *l* = 1, …, *L*, according to the subcomponent weight vector **w**_*k*_ = (*w*_*k*1_, …, *w_kL_*). The cluster-specific parameter vector
(3)
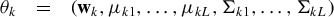
consists of **w**_*k*_ as well as the means 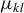 and covariance matrices 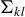 of all Gaussian subcomponent densities. *L* is typically unknown, but as we are not interested in estimating the “true” number of subcomponents *L* forming the cluster, we only ensure that *L* is chosen sufficiently large to obtain an accurate approximation of the cluster distributions. While the choice of *L* is not crucial to ensure a good model fit as long as *L* is sufficiently large, a too generous choice of *L* should be avoided for computational reasons as the computational complexity of the estimation increases with the number of subcomponents *L*.

By choosing[Fig f0001]the prior 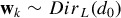 with *d*_0_ = *d*/2 + 2, the approximation of the cluster density is obtained by filling all *L* subcomponents, thus avoiding empty subcomponents. This choice is motivated again by the results of Rousseau and Mengersen (2011) who show that, if *d*_0_ > *d*/2, the posterior density asymptotically handles an overfitting mixture by splitting “true” components into two or more identical components.

### 2.2 Identification Through Hierarchical Priors

When fitting the finite mixture model (1) with semiparametric cluster densities given by (2), we face a special identifiability problem, since the likelihood is entirely agnostic about which subcomponents form a cluster. Indeed, the likelihood is completely ignorant concerning the issue which of the *K* · *L* components belong together, since (1) can be written as an expanded Gaussian mixture with *K* · *L* components with weights 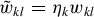,
(4)
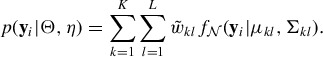
These *K* · *L* components can be permuted in (*K* · *L*)! different ways and the resulting ordering can be used to group them into *K* different cluster densities, without changing the mixture likelihood (4). Hence, the identification of (1), up to label switching on the upper level, hinges entirely on the prior distribution.

Subsequently, we suggest a hierarchical prior that addresses these issues explicitly. Conditional on a set of fixed hyperparameters φ_0_ = (*e*_0_, *d*_0_, *c*_0_, *g*_0_, **G**_0_, **B**_0_, **m**_0_, **M**_0_, ν), the weight distribution 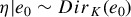 and the *K* cluster-specific parameter vectors 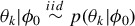 are independent a priori, that is:
(5)
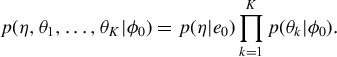
This prior formulation ensures that the *K* non-Gaussian cluster distributions of the upper level mixture (1) are invariant to permutations. We further assume that within each cluster *k*, the prior distribution 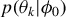 admits the following block independence structure:
(6)
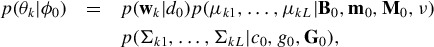
where 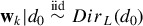. Conditional on φ_0_, the subcomponent means 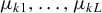 are dependent a priori as are the subcomponent covariance matrices 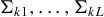. However, they are assumed to be exchangeable to guarantee that within each cluster *k*, the *L* Gaussian subcomponents in (2) can be permuted without changing the prior.

To create this dependence, a hierarchical “random effects” prior is formulated, where, on the upper level, conditional on the fixed upper level hyperparameters (*g*_0_, **G**_0_, **m**_0_, **M**_0_, ν), cluster-specific random hyperparameters (**C**_0*k*_, **b**_0*k*_), and 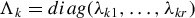, are drawn independently for each *k* = 1, …, *K* from a set of three independent base distributions:
(7)
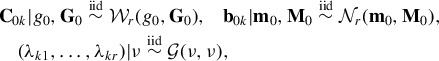
where 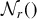 and 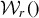 denote the *r*-multivariate normal and Wishart distribution for the parametrization see e.g. Frühwirth-Schnatter 2006, respectively, and 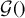 the gamma distribution, parameterized such that *E*(λ_*kl*_|ν) = 1.

On the lower level, conditional on the cluster-specific random hyperparameters (**C**_0*k*_, **b**_0*k*_, 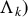 and the fixed lower level hyperparameters (**B**_0_, *c*_0_), the *L* subcomponent means 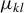 and covariance matrices 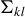 are drawn independently for all *l* = 1, …, *L*:
(8)
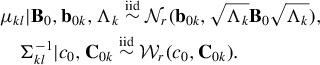



### 2.3 Tuning the Hyperparameters

To identify the mixture of mixtures model given in (1) and (2) through the prior defined in Section 2.2, the fixed hyperparameters φ_0_ have to be chosen carefully. In addition, we select them in a way to take the data scaling into account, avoiding the need to standardize the data prior to data analysis.

First, it is essential to clarify what kind of shapes and forms are aimed at as cluster distributions. We give the following (vague) characterization of a data cluster: a data cluster is a very “dense” region of data points, with possibly no “gaps” within the cluster distribution, whereas different clusters should be located well-separated from each other, that is, here large “gaps” between the cluster distributions are desired. We confine ourselves to the investigation of clusters with approximately convex cluster shapes, where the cluster center can be seen as a suitable representative for the entire cluster. Regarding volume, orientation, or asymmetry of the data clusters we are looking for, no constraints on the cluster shapes and forms are imposed.

Based on this cluster concept, our aim is to model a dense and connected cluster distribution by a mixture of normal subcomponents. Various strategies regarding the modeling of the subcomponent means and covariance matrices could be employed. We decided to allow for flexible shapes for the single subcomponents, ensuring that they strongly overlap at the same time. An alternative approach would be to use constrained simple shaped subcomponents, for example, subcomponents with identical isotropic covariance matrices. However, in this case, a large number of subcomponents might be needed to cover the whole cluster region and shrinkage of the subcomponent means toward the common cluster center may not be possible. Since then some of the subcomponents have to be located far away from the cluster center to fit also boundary points, considerable distances have to be allowed between subcomponent means. This induces the risk of gaps within the cluster distribution and a connected cluster distribution may not result. Therefore, in our approach the cluster distributions are estimated as mixtures of only a few but unconstrained, highly dispersed and heavily overlapping subcomponents where the means are strongly pulled toward the cluster center. In this way, a connected cluster distribution is ensured.

In a Bayesian framework, we need to translate these modeling purposes into appropriate choices of hyperparameters. On the upper level, the covariance matrix **M**_0_ controls the amount of prior shrinkage of the cluster centers **b**_0*k*_ toward the overall data center **m**_0_, which we specify as the midpoint of the data. To obtain a prior, where the cluster centers **b**_0*k*_ are allowed to be widely spread apart and almost no shrinkage toward **m**_0_ takes place, we choose **M**_0_ ≫ **S**_*y*_, where **S**_*y*_ is the sample covariance matrix of all data, for example **M**_0_ = 10**S**_*y*_.

**Figure 1. f0001:**
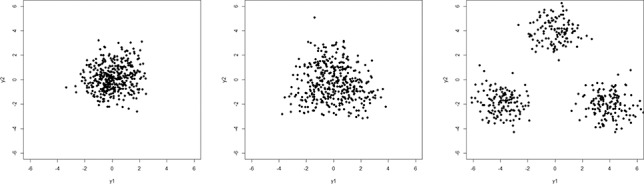
Variance decomposition of a mixture distribution. Scatterplots of samples from a standard normal mixture distribution with three components and equal weights, with a varying amount of heterogeneity φ_*B*_ explained by the variation of the component means, φ_*B*_ = 0.1, φ_*B*_ = 0.5, and φ_*B*_ = 0.9 (from left to right).

Our strategy for appropriately specifying the hyperparameters **G**_0_ and **B**_0_ is based on the variance decomposition of the mixture of mixtures model, which splits 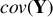 into the different sources of variation. For a finite mixture model with *K* clusters, as given in (1), the total heterogeneity 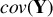 can be decomposed in the following way (Frühwirth-Schnatter 2006, p. 170):
(9)
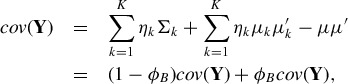
where the cluster means 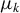 and the cluster covariance matrices 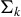 are the first and second central moments of the cluster distribution 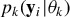 and 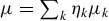 is the mixture mean. In this decomposition, φ_*B*_ is the proportion of the total heterogeneity explained by the variability of the cluster means 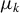 and (1 − φ_*B*_) is the proportion explained by the average variability within the clusters. The larger φ_*B*_, the more the clusters are separated, as illustrated in Figure 1 for a three-component standard Gaussian mixture with varying values of φ_*B*_.

For a mixture of mixtures model, the heterogeneity 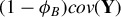 explained within a cluster can be split further into two sources of variability, namely the proportion φ_*W*_ explained by the variability of the subcomponent means 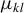 around the cluster center 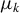, and the proportion (1 − φ_*W*_) explained by the average variability within the subcomponents:
(10)
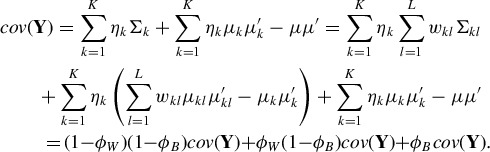
Based on this variance decomposition, we select the proportions φ_*B*_ and φ_*W*_ and incorporate them into the specification of the hyperparameters of our hierarchical prior.

φ_*B*_ defines the proportion of variability explained by the different cluster means. We suggest to specify φ_*B*_ not too large, for example, to use φ_*B*_ = 0.5. This specification may seem to be counterintuitive as to model well-separated clusters it would seem appropriate to select φ_*B*_ large. However, if φ_*B*_ is large, the major part of the total heterogeneity of the data is already explained by the variation (and separation) of the cluster means, and, as a consequence, only a small amount of heterogeneity is left for the within-cluster variability. This within-cluster variability in turn will get even more diminished by the variability explained by the subcomponent means leading to a small amount of variability left for the subcomponents. Thus for large values of φ_*B*_, estimation of tight subcomponent densities would result, undermining our modeling aims.

φ_*W*_ defines the proportion of within-cluster variability explained by the subcomponent means. φ_*W*_ also controls how strongly the subcomponent means are pulled together and influences the overlap of the subcomponent densities. To achieve strong shrinkage of the subcomponent means toward the cluster center, we select small values of φ_*W*_, for example, φ_*W*_ = 0.1. Larger values of φ_*W*_ may introduce gaps within a cluster, which we want to avoid.

Given φ_*B*_ and φ_*W*_, we specify the scale matrix **G**_0_ of the prior on **C**_0*k*_ such that the a priori expectation of the first term in the variance decomposition (10), given by

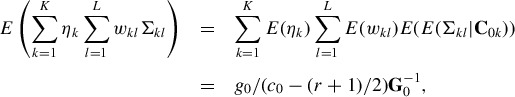
matches the desired amount of heterogeneity explained by a subcomponent:
(11)
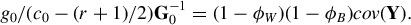
We replace 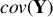 in (11) with the main diagonal of the sample covariance **S**_*y*_ to take only the scaling of the data into account (see, e.g., Frühwirth-Schnatter 2006). This gives the following specification for **G**_0_:
(12)
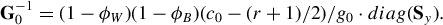
Specification of the prior of the subcomponent covariance matrices 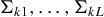 is completed by defining the scalar prior hyperparameters *c*_0_ and *g*_0_. Frühwirth-Schnatter (2006, Section 6.3.2, p. 192) suggested to set *c*_0_ > 2 + (*r* − 1)/2. In this way, the eigenvalues of 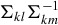 are bounded away from 0 avoiding singular matrices. We set *c*_0_ = 2.5 + (*r* − 1)/2 to allow for a large variability of 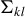. The Wishart density is regular if *g*_0_ > (*r* − 1)/2 and in the following we set *g*_0_ = 0.5 + (*r* − 1)/2.

Regarding the prior specification of the subcomponent means 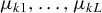, we select the scale matrix **B**_0_ to concentrate a lot of mass near the cluster center **b**_0*k*_, pulling 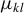 toward **b**_0*k*_. Matching the a priori expectation of the second term in the variance decomposition (10), given by

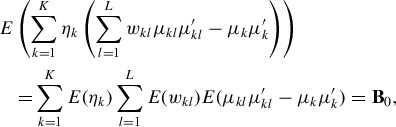
to the desired proportion of explained heterogeneity and, using once more only the main diagonal of **S**_*y*_ we obtain 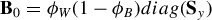, which incorporates our idea that only a small proportion φ_*W*_ of the within-cluster variability should be explained by the variability of the subcomponent means.

After having chosen φ_*B*_ and φ_*W*_, basically the cluster structure and shape is a priori determined. However, to allow for more flexibility in capturing the unknown cluster shapes in the sense that within each cluster the amount of shrinkage of the subcomponent means 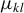 toward the cluster center **b**_0*k*_ need not to be the same for all dimensions, for each cluster *k* and each dimension *j* additionally a random adaptation factor λ_*kj*_ is introduced in (8) which adjusts **B**_0_. The gamma prior for λ_*kj*_ in (7) implies that the prior expectation of the covariance matrix of 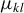 equals **B**_0_. However, λ_*kj*_ acts as a local adjustment factor for cluster *k* which allows to shrink (or inflate) the variance of subcomponent means μ_*klj*_ in dimension *j* to adapt to a more (or less) dense cluster distribution as specified by **B**_0_. To allow only for small adjustments of the specified **B**_0_, we choose ν = 10, in this way almost 90% of the a priori values of λ_*kj*_ are between 0.5 and 1.5. This hierarchical prior specification for 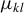 corresponds to the normal gamma prior (Griffin and Brown 2010) which has been applied by Frühwirth-Schnatter (2011) and Malsiner-Walli, Frühwirth-Schnatter, and Grün (2016) in the context of finite mixture models for variable selection.

### 2.4 Relation to BNP Mixtures

Our approach bears resemblance to various approaches in BNP modeling. First of all, the concept of sparse finite mixtures as used in Malsiner-Walli, Frühwirth-Schnatter, and Grün (2016) is related to Dirichlet process (DP) mixtures (Müller and Mitra 2013) where the discrete mixing distribution in the finite mixture (1) is substituted by a random distribution 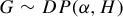, drawn from a DP prior with precision parameter α and base measure *H*. As a draw *G* from a DP is almost surely discrete, the corresponding model has a representation as an infinite mixture:
(13)
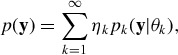
with iid atoms 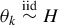 drawn from the base measure *H* and weights η_*k*_ = *v_k_*∏^*k* − 1^_*j* = 1_(1 − *v_j_*) obeying the stick breaking representation with 
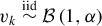
 (Sethuraman 1994).

If the hyperparameter in the weight distribution 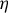 of a sparse finite mixture is chosen as *e*_0_ = α/*K*, that is 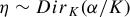, and the component parameters 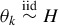 are iid draws from *H*, then as *K* increases, the sparse finite mixture in Equation (1) converges to a DP mixture with mixing distribution 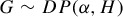, see Green and Richardson (2001). For example, the sparse finite Gaussian mixture introduced in Malsiner-Walli, Frühwirth-Schnatter, and Grün (2016) converges to a Dirichlet process Gaussian mixture as *K* increases, with 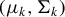 being iid draws from the appropriate base measure *H*.

The more general sparse finite mixture of mixtures model introduced in this article also converges to a Dirichlet process mixture, where the atoms are finite mixtures indexed by the parameter 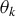 defined in (3). The parameters 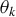 are iid draws from the base measure (6), with strong dependence among the means 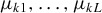 and covariances 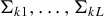 within each cluster *k*. This dependence is achieved through the two-layer hierarchical prior described in (7) and (8) and is essential to create well-connected clusters from the subcomponents, as outlined in Section 2.3.

Also in the BNP framework models have been introduced that create dependence, either in the atoms and/or in the weights attached to the atoms. For instance, the nested DP process of Rodriguez, Dunson, and Gelfand (2008) allows us to cluster distributions across *N* units. Within each unit *i*, *i* = 1, …, *N*, repeated (univariate) measurements *y_it_*, *t* = 1, …, *N_i_* arise as independent realizations of a DP Gaussian mixture with random mixing distribution *G_i_*. The *G_i_*'s are iid draws from a DP, in which the base measure is itself a Dirichlet process 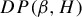, that is, 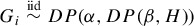. Hence, two distributions *G_i_* and *G_j_* either share the same weights and atoms sampled from *H*, or the weights and atoms are entirely different. If only a single observation *y_i_* is available in each unit, that is, *N_i_* = 1, then the nested DP is related to our model. In particular, it has a two-layer representation as in (1) and (2), however, with both *K* and *L* being infinite. The nested DP can, in principal, be extended to multivariate observations **y**_*i*_. In this case, *p*(**y**_*i*_) takes the same form as in (13), with the same stick breaking representation for the cluster weights η_1_, η_2_, …. On the lower level, each cluster distribution 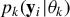 is a DP Gaussian mixture:
(14)
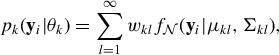
where the component weights *w_kl_* are derived from the stick breaking representation *w_kl_* = *u_kl_*∏^*l* − 1^_*j* = 1_(1 − *u_kj_*), *l* = 1, 2, … where 
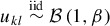
. For the nested DP, dependence is introduced only on the level of the weights and sticks, as the component parameters 
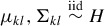
 are iid draws from the base measure *H*. This lack of prior dependence among the atoms 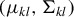 is likely to be an obstacle in a clustering context.

The BNP approach most closely related to our model is the infinite mixture of infinite Gaussian mixtures (I^2^GMM) model of Yerebakan, Rajwa, and Dundar (2014) which also deals with clustering multivariate observations from non-Gaussian component densities.^1^ 1We would like to thank a reviewer for pointing us to this article. The I^2^GMM model has a two-layer hierarchical representation like the nested DP. On the top level, iid cluster-specific locations **b**_0*k*_ and covariances 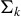 are drawn from a random distribution 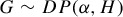 arising from a DP prior with base measure *H* being equal to the conjugate normal-inverse-Wishart distribution. A cluster-specific DP is introduced on the lower level as for the nested DP; however, the I^2^GMM model is more suited for clustering, as prior dependence is also introduced among the atoms belonging to the same cluster. More precisely, 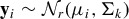, with 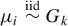, where 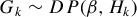 is a draw from a DP with cluster-specific base measure 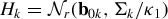.

It is easy to show that the I^2^GMM model has an infinite two-layer representation as in (13) and (14), with exactly the same stick breaking representation.^2^ 2Note that the notation in Yerebakan, Rajwa, and Dundar (2014) is slightly different, with γ and α corresponding to α and β introduced above. However, the I^2^GMM model has a constrained form on the lower level, with homoscedastic covariances 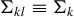, whereas the locations 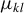 scatter around the cluster centers **b**_0*k*_ as in our model:
(15)
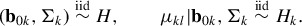
In our sparse mixture of mixtures model, we found it useful to base the density estimator on heteroscedastic covariances 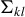, to better accommodate the non-Gaussianity of the cluster densities with a fairly small number *L* of subcomponents. It should be noted that our semiparametric density estimator is allowed to display nonconvex shapes, as illustrated in Figure C.2 in the Appendix. Nevertheless, we could have considered a mixture in (2) where 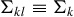, with the same base measure for the atoms 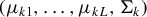 as in (15). In this case, the relationship between our sparse finite mixture and the I^2^GMM model would become even more apparent: by choosing *e*_0_ = α/*K* and *d*_0_ = β/*L* and letting *K* and *L* go to infinity, our model would converge to the I^2^GMM model.

## 3. Clustering and Posterior Inference

### 3.1 Clustering and Selecting the Number of Clusters

For posterior inference, two sequences of allocation variables are introduced, namely the cluster assignment indicators **S** = (*S*_1_, …, *S_N_*) and the within-cluster allocation variables **I** = (*I*_1_, …, *I_N_*). More specifically, *S_i_* ∈ {1, …, *K*} assigns each observation **y**_*i*_ to cluster *S_i_* on the upper level of the mixture of mixtures model. On the lower level, *I_i_* ∈ {1, …, *L*} assigns observation **y**_*i*_ to subcomponent *I_i_*. Hence, the pair (*S_i_*, *I_i_*) carries all the information needed to assign each observation to a unique component in the expanded mixture (4).

Note that for all observations **y**_*i*_ and **y**_*j*_ belonging to the same cluster, the upper level indicators *S_i_* = *S_j_* will be the same, while the lower level indicators *I_i_* ≠ *I_j_* might be different, meaning that they belong to different subcomponents within the same cluster. It should be noted that the Dirichlet prior 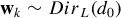, with *d*_0_ > *d*/2, on the weight distribution ensures overlapping densities within each cluster, in particular if *L* is overfitting. Hence, the indicators *I_i_* will typically cover all possible values {1, …, *L*} within each cluster.

For clustering, only the upper level indicators **S** are explored, integrating implicitly over the uncertainty of assignment to the subcomponents on the lower level. A cluster *C_k_* = {*i*|*S_i_* = *k*} is thus a subset of the data indices {1, …, *N*}, containing all observations with identical upper level indicators. Hence, the indicators **S** define a random partition 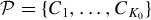 of the *N* data points in the sense of Lau and Green (2007), as **y**_*i*_ and **y**_*j*_ belong to the same cluster, if and only if *S_i_* = *S_j_*. The partition 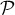 contains 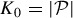 clusters, where 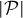 is the cardinality of 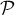. Due to the Dirichlet prior 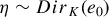, with *e*_0_ close to 0 to obtain a sparse finite mixture, *K*_0_ is a random number being a priori much smaller than *K*.

For a sparse finite mixture model with *K* clusters, the prior distribution over all random partitions 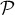 of *N* observations is derived from the joint (marginal) prior *p*(**S**) = ∫∏^*N*^_*i* = 1_ *p*(*S_i_*|***η***)*p*(***η***)*d* ***η*** which is given, for example, in Frühwirth-Schnatter (2006, p. 66):
(16)
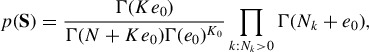
where 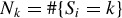. For a given partition 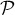 with *K*_0_ data clusters, there are *K*!/(*K* − *K*_0_)! assignment vectors **S** that belong to the equivalence class defined by 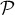. The prior distribution over all random partitions 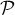 is then obtained by summing over all assignment vectors **S** that belong to the equivalence class defined by 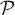:
(17)
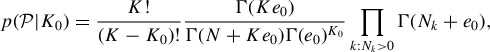
which takes the form of a product partition model and therefore is invariant to permuting the cluster labels. Hence, it is possible to derive the prior predictive distribution *p*(*S_i_*|**S**_− *i*_), where **S**_− *i*_ denote all indicators, excluding *S_i_*. Let *K* ^− *i*^_0_ be the number of nonempty clusters implied by **S**_− *i*_ and let *N* ^− *i*^_*k*_ be the corresponding cluster sizes. From (16), we obtain the following probability that *S_i_* is assigned to an existing cluster *k*:
(18)
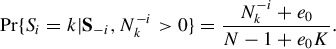
The prior probability that *S_i_* creates a new cluster with *S_i_* ∈ *I* = {*k*|*N* ^− *i*^_*k*_ = 0} is equal to
(19)
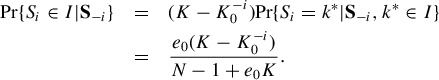
It is illuminating to investigate the prior probability to create new clusters in detail. First of all, for *e*_0_ independent of *K*, this probability not only depends on *e*_0_, but also increases with *K*. Hence, a sparse finite mixture model based on the prior 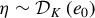 can be regarded as a two-parameter model, where both *e*_0_ and *K* influence the a priori expected number of data clusters *K*_0_ which is determined for a DP mixture solely by α. A BNP two-parameter mixture is obtained from the Pitman–Yor process (PYP) prior 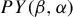 with β ∈ [0, 1), and α > −β (Pitman and Yor 1997), with stickbreaking representation 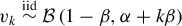. The DP prior results as that special case where β = 0.

Second, the prior probability (19) to create new clusters in a sparse finite mixture model decreases, as the number *K* ^− *i*^_0_ of nonempty clusters increases. This is in sharp contrast to DP mixtures where this probability is constant and PYP mixtures, where this probability increases, see, for example, Fall and Barat (2014).

Finally, what distinguishes a sparse finite mixture model, both from a DP as well as a PYP mixture, is the a priori expected number of data clusters *K*_0_, as the number *N* of observations increases. For *K* and *e*_0_ independent of *N*, the probability to create new clusters decreases, as *N* increases, and converges to 0, as *N* goes to infinity. Therefore, *K*_0_ is asymptotically independent of *N* for sparse finite mixtures, whereas for the DP process *K*_0_ ∼ αlog (*N*) (Korwar and Hollander 1973) and *K*_0_ ∼ *N* ^β^ obeys a power law for PYP mixtures (Fall and Barat 2014). This leads to quite different clustering behavior for these three types of mixtures.

A well-known limitation of DP priors is that a priori the cluster sizes are expected to be geometrically ordered, with one big cluster, geometrically smaller clusters, and many singleton clusters (Müller and Mitra 2013). PYP mixtures are known to be more useful than the DP mixture for data with many significant, but small clusters. A common criticism concerning finite mixtures is that the number of clusters needs to be known a priori. Since this is not the case for sparse finite mixtures, they are useful in the context of clustering, in particular in cases where the data arise from a moderate number of clusters, that does not increase as the number of data points *N* increases.

### 3.2 MCMC Estimation and Posterior Inference

Bayesian estimation[Table T0001]of the sparse hierarchical mixture of mixtures model is performed using MCMC methods based on data augmentation and Gibbs sampling. We only need standard Gibbs sampling steps, see the detailed MCMC sampling scheme in Appendix A.

To perform inference based on the MCMC draws, that is, to cluster the data, to estimate the number of clusters, to solve the label switching problem on the higher level and to estimate cluster-specific parameters, several existing procedures can be easily adapted and applied to post-process the posterior draws of a mixture of mixtures model, for example, those which are, for instance, implemented in the R packages PReMiuM (Liverani et al. 2015) and label.switching (Papastamoulis 2015).

For instance, the approach in PReMiuM is based on the posterior probabilities of co-clustering, expressed through the similarity matrix Pr{*S_i_* = *S_j_*|**y**} which can be estimated from the *M* posterior draws **S** ^(*m*)^, *m* = 1, …, *M*, see Appendix B for details. The methods implemented in label.switching aim at resolving the label switching problem when fitting a finite mixture model using Bayesian estimation. Note that in the case of the mixture of mixtures model label switching occurs on two levels. On the cluster level, the label switching problem is caused by invariance of the mixture likelihood given in Equation (1) with respect to reordering of the clusters. On this level, label switching has to be resolved, since the single cluster distributions need to be identified. On the subcomponent level, label switching happens due to the invariance of Equation (2) with respect to reordering of the subcomponents. As we are only interested in estimating the entire cluster distributions, it is not necessary to identify the single subcomponents. Therefore, the label switching problem can be ignored on this level.

In this article, the post-processing approach employed first performs a model selection step. The posterior draws of the indicators **S** ^(*m*)^, *m* = 1, …, *M* are used to infer the number of nonempty clusters *K* ^(*m*)^_0_ on the upper level of the mixture of mixtures model and the number of data clusters is then estimated as the mode. Conditional on the selected model, an identified model is obtained based on the point process representation of the estimated mixture. This method was introduced in Frühwirth-Schnatter (2006, p. 96) and successfully applied to model-based clustering in various applied research, see, for example, Frühwirth-Schnatter (2011) for some review. This procedure was adapted to sparse finite mixtures by Frühwirth-Schnatter (2011) and Malsiner-Walli, Frühwirth-Schnatter, and Grün (2016) and is easily extended to deal with sparse mixture of mixtures models, see Appendix B for more details. We will use this post-processing approach in our simulation studies and the applications in Section 4 and Appendices C, D, and F to determine a partition of the data based on the maximum a posteriori (MAP) estimates of the relabeled cluster assignments.

## 4. Simulation Studies and Applications

The performance of the proposed strategy for selecting the unknown number of clusters and identifying the cluster distributions is illustrated in two simulation studies. In the first simulation study, we investigate whether we are able to capture dense non-Gaussian data clusters and estimate the true number of data clusters. Furthermore, the influence of the specified maximum number of clusters *K* and subcomponents *L* on the clustering results is studied. In the second simulation study, the sensitivity of the a priori defined proportions φ_*B*_ and φ_*W*_ on the clustering result is investigated. For a detailed description of the simulation design and results, see Appendix C. Overall, the results indicated that our approach performed well and yielded promising results.

To further evaluate our approach, we fit the sparse hierarchical mixture of mixtures model on benchmark datasets and real data. First, we consider five datasets which were previously used to benchmark algorithms in cluster analysis. For these datasets, we additionally apply the “merging strategy” proposed by Baudry et al. (2010) to compare the results to those of our approach. For these benchmark datasets, class labels are available and we assess the performance by comparing how well our approach is able to predict the class labels using the cluster assignments, measured by the misclassification rate as well as the adjusted Rand index.

To assess how the algorithm scales to larger datasets, we investigate the application to two flow cytometry datasets. The three-dimensional DLBCL dataset (Lee and McLachlan 2013) consists of around 8000 observations and comes with manual class labels which can be used as benchmark. The GvHD dataset (Brinkman et al. 2007) consists of 12,441 observations, but no class labels are available. We compare the clusters detected for this dataset qualitatively to solutions previously reported in the literature.

The detailed[Fig f0002]description of all[Fig f0003]investigated datasets as well as of the derivation of the performance measures are given in Appendix D. For the benchmark datasets, the number of estimated clusters 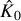, the adjusted Rand index (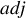), and misclassification rate (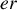) are reported in Table 1 for all estimated models. In the first columns of Table 1, the name of the dataset, the number of observations *N*, the number of variables *r* and the number of true classes 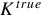 (if known) are reported. To compare our approach to the merging approach proposed by Baudry et al. (2010), we use the function Mclust of the R package mclust (Fraley et al. 2012) to first fit a standard normal mixture distribution with the maximum number of components *K* = 10. The number of estimated normal components based on the BIC is reported in the column Mclust. Then, the selected components are combined hierarchically to clusters by calling function clustCombi from the same package (column clustCombi). The number of clusters is chosen by visual detection of the change point in the plot of the rescaled differences between successive entropy values, as suggested by Baudry et al. (2010). Furthermore, to compare our results to those obtained if a cluster distribution is modeled by a single normal distribution only, a sparse finite mixture model with *K* = 10 (Malsiner-Walli, Frühwirth-Schnatter, and Grün 2016) is fitted to the datasets (column *SparseMix*). The results of fitting a sparse hierarchical mixture of mixtures model with *K* = 10 are given in column *SparseMixMix*, where *L* = 5 is compared to our default choice of *L* = 4 to investigate robustness with respect to the choice of *L*. For each estimation, MCMC sampling is run for 4000 iterations after a burn-in of 4000 iterations.

**Table 1. T0001:** Results for the estimated number of data clusters 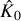 for various benchmark datasets, using the functions Mclust to fit a standard mixture model with *K* = 10 and clustCombi to estimate a mixture with combined components (column *Mclust*), using a sparse finite mixture model with *K* = 10 (column *SparseMix*), and estimating a sparse hierarchical mixture of mixtures model with *K* = 10, φ_*B*_ = 0.5 and φ_*W*_ = 0.1, and *L* = 4, 5 (column *SparseMixMix*). Priors and hyperparameter specifications are selected as described in Section 2. In parentheses, the adjusted Rand index (“1” corresponds to perfect classification) and the proportion of misclassified observations (“0” corresponds to perfect classification) are reported.

				*Mclust*		*SparseMix*	*SparseMixMix*	
				*K* = 10		*K* = 10	*K* = 10	
Dataset	*N*	*r*		Mclust	clustCombi	*L* = 1	*L* = 4	*L* = 5
Yeast	626	3	2	8 *(0.50, 0.20)*	6 *(−0.02, 0.25)*	6 *(0.48, 0.23)*	**2***(0.68, 0.08)*	2 *(0.71, 0.07)*
Flea beetles	74	6	3	5 *(0.77, 0.18)*	4 *(0.97, 0.03)*	3 *(1.00, 0.00)*	**3***(1.00, 0.00)*	3 *(1.00, 0.00) *
AIS	202	3	2	3 *(0.73, 0.13)*	2 *(0.66, 0.09)*	3 *(0.76, 0.11)*	**2***(0.81, 0.05*)	2 *(0.76, 0.06)*
Wisconsin	569	3	2	4 *(0.55, 0.30)*	4 *(0.55, 0.30*)	4 *(0.62, 0.21)*	**2***(0.82, 0.05)*	2 *(0.82, 0.05)*
Flower	400	2	4	6 *(0.52, 0.35)*	4 *(0.99, 0.01)*	5 *(0.67, 0.20)*	**4***(0.97, 0.01)*	4 *(0.97, 0.02)*

As can be seen in Table 1, for all datasets the sparse hierarchical mixture of mixtures model is able to capture the data clusters quite well both in terms of the estimated number of clusters and the clustering quality measured by the misclassification rate as well as the adjusted Rand index. In general, our approach is not only outperforming the standard model-based clustering model using mixtures of Gaussians regarding both measures, but also the approach proposed by Baudry et al. (2010). In addition, it can be noted that for all datasets the estimation results remain quite stable, if the number of subcomponents *L* is increased to 5, see the last column in Table 1. The results for the Yeast dataset are of particular interest as they indicate that clustCombi completely fails. Although the misclassification rate of 25% implies that only a quarter of the observations is assigned to “wrong” clusters, inspection of the clustering obtained reveals that almost all observations are lumped together in a single, very large cluster, whereas the few remaining observations are split into five very small clusters. This bad clustering quality is better reflected by the adjusted Rand index which takes a negative value (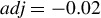), is “worse than would be expected by guessing” (Franczak, Browne, and McNicholas 2012). For the flower dataset, more results are given in Appendix D, where the obtained clustering and cluster distributions are illustrated.

To investigate the performance of our approach on larger datasets, we fit the sparse hierarchical mixture of mixtures model to two flow cytometry datasets. These applications also allow us to indicate how the prior settings need to be adapted if a different cluster structure is assumed to be present in the data. As generally known, flow cytometry data exhibit non-Gaussian characteristics such as skewness, multimodality, and a large number of outliers, as can be seen in the scatterplot of two variables of the GvHD dataset in Figure 3.

Thus, we specified a sparse hierarchical mixture of mixtures model with *K* = 30 clusters and increased the number of subcomponents forming a cluster to *L* = 15 to handle more complex shapes of the cluster distributions given the large amount of data. Since the flow cytometry data clusters have a lot of outliers similar to the clusters generated by shifted asymmetric Laplace (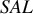) distributions (see Appendix F), we substitute the hyperprior 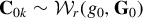 by the fixed value **C**_0*k*_ = *g*_0_ **G** ^− 1^_0_ and set λ_*kj*_ ≡ 1, *j* = 1, …, *r* to prevent that within a cluster the subcomponent covariance matrices are overly shrunken and become too similar. In this way, subcomponent covariance matrices are allowed to vary considerably within a cluster and capture both a dense cluster region around the cluster center and scattered regions at the boundary of the cluster. We fit this sparse hierarchical mixture of mixtures model to the DLBCL data after removing 251 dead cells.

**Figure 2. f0002:**
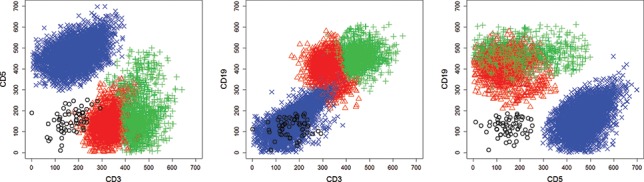
Flow cytometry dataset DLBCL. Scatterplot of the clustering results.

For most MCMC runs after a few hundred iterations, all but four clusters become empty during MCMC sampling. The estimated four cluster solution coincides almost exactly with the cluster solution obtained with manual gating; the adjusted Rand index is 0.95 and the error rate equals 0.03. This error rate outperforms the error rate of 0.056 reported by Lee and McLachlan (2013). In Figure 2, the estimated four cluster solution is visualized.

**Figure 3. f0003:**
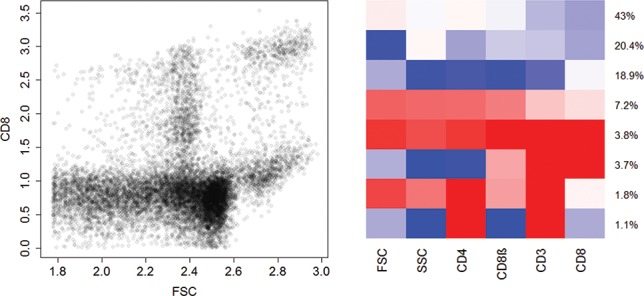
Flow cytometry dataset GvHD. Scatterplot of two variables (“FSC,” “CD8”) (left-hand side), and heatmap of the clustering results by fitting a sparse hierarchical mixture of mixtures model (right-hand side). In the heatmap, each row represents the location of a six-dimensional cluster, and each column represents a particular marker. The red, white, and blue colors denote high, medium, and low expression, respectively.

When fitting a sparse hierarchical mixture of mixtures model to the GvHD data, the classifications resulting from different runs of the MCMC algorithm seem to be rather stable. The obtained solutions differ mainly in the size of the two large clusters with low expressions. These, however, are supposed to not contain any information regarding the development of the disease. On the right-hand side of Figure 3, the results of one specific run are shown in a heatmap. In this run, we found eight clusters which are similar to those reported by Frühwirth-Schnatter and Pyne (2010) when fitting a skew-*t* mixture model to these data. In the heatmap, each row represents the location of a six-dimensional cluster, and each column represents a particular marker (variable). The red, white, and blue colors denote high, medium, and low expressions.

As in Frühwirth-Schnatter and Pyne (2010), we identified two larger clusters (43% and 20.4%, first two rows in the heatmap) with rather low expressions in the last four variables. We also identified a smaller cluster (3.8%, forth row from the bottom) representing live cells (high values in the first two variables) with a unique signature in the other four variables (high values in all four variables). Also, two other small clusters can be identified (second and third rows from the bottom) which have a signature very similar to the clusters found by Frühwirth-Schnatter and Pyne (2010), and thus our results confirm their findings.

## 5. Discussion

We propose suitable priors for fitting an identified mixture of normal mixtures model within the Bayesian framework of model-based clustering. This approach allows for (1) automatic determination of the number of clusters and (2) semi-parametric approximation of non-Gaussian cluster distributions by mixtures of normals. We only require the assumption that the cluster distributions are dense and connected. Our approach consists in the specification of structured informative priors on all model parameters. This imposes a rigid hierarchical structure on the normal subcomponents and allows for simultaneous estimation of the number of clusters and their approximating distributions. This is in contrast to the two-step merging approaches, where in the first step the data distribution is approximated by a suitable normal mixture model. However, because this approximation is made without taking the data clusters into account which are reconstructed only in the second step of the procedure, the general cluster structure might be missed by these approaches.

As we noted in our simulation studies, the way in which the cluster mixture distributions are modeled by the subcomponent densities is crucial for the clustering result. Enforcing overlapping subcomponent densities is essential to avoid that a single subcomponent becomes too narrow thus leading to a small a posteriori cluster probability for observations from this subcomponent. Also, enforcing that observations are assigned to *all* subcomponents during MCMC sampling is important as the estimation of empty subcomponents would bias the resulting cluster distribution because of the “prior” subcomponents. For modeling large, overlapping subcomponent densities, crucial model parameters are the a priori specified covariance matrix of the subcomponent means and the scale matrix of the inverse Wishart prior for the subcomponent covariance matrices. We select both crucial hyperparameters based on the variance decomposition of a mixture of mixtures model.

We found a prior setting which is able to capture dense and connected data clusters in a range of benchmark datasets. However, if interest lies in detection of different cluster shapes, a different tuning of the prior parameters may be required. Therefore, it would be interesting to investigate in more detail how we can use certain prior settings to estimate specific kinds of data clusters. Then, it would be possible to give recommendations which prior settings have to be used to capture certain types of data clusters. For instance, mixtures of shifted asymmetric Laplace (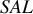) distributions, introduced by Franczak, Browne, and McNicholas (2012), have cluster distributions which are nondense and have a strongly asymmetric shape with comet-like tails. In this case, the prior specifications given in Section 2 are not able to capture the clusters and need to be tuned to capture this special kind of data clusters, see the example given in Appendix F.

Although our approach to estimate the number of clusters worked well for many datasets, we encountered mixing problems with the blocked conditional Gibbs sampler outlined in Appendix A, in particular in high-dimensional spaces with large datasets. To alleviate this problem, a collapsed sampler similar to Fall and Barat (2014) could be derived for finite mixtures. However, we leave this for future research.

## Supplementary Material

Supplementary Materials

## Supplementary Materials

**Appendix** containing (A) the MCMC scheme to estimate a mixture of mixtures model, (B) a detailed description of the post-processing strategy based on the point process representation, (C) the simulation studies described in Section 4, (D) a description of the datasets studied in Section 4, (E) an illustration of issues with the merging approach, and (F) estimation of data clusters generated by a -distribution (Franczak, Browne, and McNicholas 2012). (Appendix.pdf)
**R Code** implementing the sparse hierarchical mixture of mixtures model (Code.zip).
