# Supplementary material for: Identifying Mixtures of Mixtures Using Bayesian Estimation
Source: J Comput Graph Stat. 2017 Apr 24;26(2):285–95. doi: 10.1080/10618600.2016.1200472 (PMC5455957; doi:10.1080/10618600.2016.1200472)
Supplement: Supplementary Materials [file ucgs_a_1200472_sm9526.zip › MixOfMix_Supp_revision-2.pdf]

Appendix to “Identifying Mixtures of Mixtures Using Bayesian Estimation”

Gertraud Malsiner-Walli
Department of Applied Statistics, Johannes Kepler University Linz
and
Sylvia Frühwirth-Schnatter
Institute of Statistics and Mathematics, Wirtschaftsuniversität Wien
and
Bettina Grün
Department of Applied Statistics, Johannes Kepler University Linz

May 31, 2016

A MCMC sampling scheme

Estimation of a sparse hierarchical mixture of mixtures model is performed through MCMC sampling based on data augmentation and Gibbs sampling. To indicate the cluster to which each observation belongs, latent allocation variables $\mathbf{S} = (S_1, \ldots, S_N)$ taking values in $\{1, \ldots, K\}^N$ are introduced such that

$$p(\mathbf{y}_i|\boldsymbol{\theta}_1, \ldots, \boldsymbol{\theta}_K, S_i = k) = p_k(\mathbf{y}_i|\boldsymbol{\theta}_k), \quad \text{and} \quad \Pr\{S_i = k|\boldsymbol{\eta}\} = \eta_k.$$

Additionally, to indicate the subcomponent to which an observation within a cluster is assigned to, latent allocation variables $\mathbf{I} = (I_1, \ldots, I_N)$ taking values in $\{1, \ldots, L\}^N$ are introduced such that

$$p_k(\mathbf{y}_i|\boldsymbol{\theta}_k, S_i = k, I_i = l) = f_{\mathcal{N}}(\mathbf{y}_i|\boldsymbol{\mu}_{kl}, \boldsymbol{\Sigma}_{kl}) \quad \text{and} \quad \Pr\{I_i = l|S_i = k, \mathbf{w}_k\} = w_{kl}.$$

Based on the priors specified in Section 2.2, with fixed hyperparameters $(e_0, d_0, c_0, g_0, \mathbf{G}_0, \mathbf{B}_0, \mathbf{m}_0, \mathbf{M}_0, \nu)$, the latent variables $(\mathbf{S}, \mathbf{I})$ and parameters $(\boldsymbol{\eta}, \mathbf{w}_k, \boldsymbol{\mu}_{kl}, \boldsymbol{\Sigma}_{kl}$, $\mathbf{C}_{0k}$, $\mathbf{b}_{0k}$, $\lambda_{kj})$, $k = 1, \ldots, K$, $l = 1, \ldots, L$, $j = 1, \ldots, r$, are sampled from the posterior

distribution using the following Gibbs sampling scheme. Note that the conditional distributions given do not indicate that conditioning is also on the fixed hyperparameters.

(1) Sampling steps on the level of the cluster distribution:

(a) *Parameter simulation step* conditional on the classifications $\mathbf{S}$. Sample $\boldsymbol{\eta}|\mathbf{S}$ from $Dir(e_1, \ldots, e_K)$, $e_k = e_0 + N_k$, $k = 1, \ldots, K$, where $N_k = \#\{S_i|S_i = k\}$ is the number of observations allocated to cluster k.

(b) *Classification step* for each observation $\mathbf{y}_i$ conditional on cluster-specific parameters. For each $i = 1, \ldots, N$ sample the cluster assignment S_i from

$$\Pr\{S_i = k|\mathbf{y}_i, \boldsymbol{\theta}, \boldsymbol{\eta}\} \propto \eta_k p_k(\mathbf{y}_i|\boldsymbol{\theta}_k), \; k = 1, \ldots, K, \tag{A.1}$$

where $p_k(\mathbf{y}_i|\boldsymbol{\theta}_k)$ is the semi-parametric mixture approximation of the cluster density:

$$p_k(\mathbf{y}_i|\boldsymbol{\theta}_k) = \sum_{l=1}^{L} w_{kl} f_{\mathcal{N}}(\mathbf{y}_i|\boldsymbol{\mu}_{kl}, \boldsymbol{\Sigma}_{kl}).$$

Note that clustering of the observations is performed on the upper level of the model, using a collapsed Gibbs step, where the latent, within-cluster allocation variables $\mathbf{I}$ are integrated out.

(2) Within each cluster k, $k = 1, \ldots, K$:

(a) *Classification step* for all observations $\mathbf{y}_i$, assigned to cluster k (i.e. $S_i = k$), conditional on the subcomponent weights and the subcomponent-specific parameters. For each $i \in \{i = 1, \ldots, N : S_i = k\}$ sample I_i from

$$\Pr\{I_i = l|\mathbf{y}_i, \boldsymbol{\theta}_k, S_i = k\} \propto w_{kl} f_{\mathcal{N}}(\mathbf{y}_i|\boldsymbol{\mu}_{kl}, \boldsymbol{\Sigma}_{kl}), \; l = 1, \ldots, L.$$

(b) *Parameter simulation step* conditional on the classifications $\mathbf{I}$ and $\mathbf{S}$:

i. Sample $\mathbf{w}_k|\mathbf{I}, \mathbf{S}$ from $Dir(d_{k1}, \ldots, d_{kL})$, $d_{kl} = d_0 + N_{kl}$, $l = 1, \ldots, L$, where $N_{kl} = \#\{I_i = l|S_i = k\}$ is the number of observations allocated to subcomponent l in cluster k.

ii. For $l = 1, \ldots, L$: Sample $\boldsymbol{\Sigma}_{kl}^{-1}|\mathbf{S}, \mathbf{I}, \boldsymbol{\mu}_{kl}, \mathbf{C}_{0k}, \mathbf{y} \sim \mathcal{W}_r(c_{kl}, \mathbf{C}_{kl})$, where

$$
\begin{aligned}
c_{kl} &= c_0 + N_{kl}/2, \\
\mathbf{C}_{kl} &= \mathbf{C}_{0k} + \frac{1}{2} \sum_{i:I_i=l,S_i=k} (\mathbf{y}_i - \boldsymbol{\mu}_{kl})(\mathbf{y}_i - \boldsymbol{\mu}_{kl})'.
\end{aligned}
$$

iii. For $l = 1, \ldots, L$: Sample $\boldsymbol{\mu}_{kl}|\mathbf{S}, \mathbf{I}, \mathbf{b}_{0k}, \boldsymbol{\Sigma}_{kl}, \boldsymbol{\Lambda}_k, \mathbf{y} \sim \mathcal{N}_r(\mathbf{b}_{kl}, \mathbf{B}_{kl})$, where

$$
\begin{aligned}
\mathbf{B}_{kl} &= (\tilde{\mathbf{B}}_{0k}^{-1} + N_{kl}\boldsymbol{\Sigma}_{kl}^{-1})^{-1}, \\
\mathbf{b}_{kl} &= \mathbf{B}_{kl}(\tilde{\mathbf{B}}_{0k}^{-1}\mathbf{b}_{0k} + \boldsymbol{\Sigma}_{kl}^{-1} N_{kl}\bar{\mathbf{y}}_{kl}),
\end{aligned}
$$

with $\tilde{\mathbf{B}}_{0k} = \sqrt{\boldsymbol{\Lambda}_k}\mathbf{B}_0\sqrt{\boldsymbol{\Lambda}_k}$, $\boldsymbol{\Lambda}_k = \text{diag}(\lambda_{k1}, \ldots, \lambda_{kr})$, and $\bar{\mathbf{y}}_{kl} = 1/N_{kl} \sum\limits_{i:I_i=l,S_i=k} \mathbf{y}_i$ being equal to the subcomponent mean for $N_{kl} > 0$ and $N_{kl}\bar{\mathbf{y}}_{kl} = 0$, otherwise.

(3) For each cluster k, $k = 1, \ldots, K$: Sample the random hyperparameters λ_{kj}, $\mathbf{C}_{0k}, \mathbf{b}_{0k}$ from their full conditionals:

(a) For $j = 1, \ldots, r$: Sample $\lambda_{kj}|\mathbf{b}_{0k}, \boldsymbol{\mu}_{k1}, \ldots, \boldsymbol{\mu}_{kL} \sim \mathcal{GIG}(p_{kL}, a_{kj}, b_{kj})$, where $\mathcal{GIG}$ is the generalized inverted Gaussian distribution and

$$
\begin{aligned}
p_{kL} &= -L/2 + \nu, \\
a_{kj} &= 2\nu, \\
b_{kj} &= \sum_{l=1}^{L} (\mu_{kl,j} - b_{0k,j})^2 / B_{0,jj}.
\end{aligned}
$$

(b) Sample $\mathbf{C}_{0k}|\boldsymbol{\Sigma}_{k1}, \ldots, \boldsymbol{\Sigma}_{kL} \sim \mathcal{W}_r(g_0 + Lc_0, \mathbf{G}_0 + \sum_{l=1}^{L} \boldsymbol{\Sigma}_{kl}^{-1})$.

(c) Sample $\mathbf{b}_{0k}|\tilde{\mathbf{B}}_{0k}, \boldsymbol{\mu}_{k1}, \ldots, \boldsymbol{\mu}_{kL} \sim \mathcal{N}_r(\tilde{\mathbf{m}}_k, \tilde{\mathbf{M}}_k)$, where

$$
\begin{aligned}
\tilde{\mathbf{M}}_k &= (\mathbf{M}_0^{-1} + L\tilde{\mathbf{B}}_{0k}^{-1})^{-1}, \\
\tilde{\mathbf{m}}_k &= \tilde{\mathbf{M}}_k \left(\mathbf{M}_0^{-1}\mathbf{m}_0 + \tilde{\mathbf{B}}_{0k}^{-1} \sum_{l=1}^{L} \boldsymbol{\mu}_{kl} \right).
\end{aligned}
$$

B Identification through clustering in the point process representation

Various post-processing approaches have been proposed for the MCMC output of finite or infinite mixture models (see, for example, Molitor et al. 2010 or Jasra et al. 2005). We pursue an approach which aims at determining a unique labeling of the MCMC draws after selecting a suitable number of clusters in order to base any posterior inference on the relabeled draws, such as for example the determination of cluster assignments.

To obtain a unique labeling of the clusters, Frühwirth-Schnatter (2006) suggested to post-process the MCMC output by clustering a vector-valued functional $f(\boldsymbol{\theta}_k)$ of the cluster-specific parameters $\boldsymbol{\theta}_k$ in the point process representation. The point process representation has the advantage that it allows to study the posterior distribution of cluster-specific parameters regardless of potential label switching, which makes it very useful for identification.

If the number K of components matches the true number of clusters, it can be expected that the vector-valued functionals of the posterior draws cluster around the "true" points $\{f(\boldsymbol{\theta}_1), \ldots, f(\boldsymbol{\theta}_K)\}$ (Frühwirth-Schnatter, 2006, p. 96). However, in the case of an overfitting mixture where draws are sampled from empty components, the clustering procedure has to be adapted as suggested in Frühwirth-Schnatter (2011) and described in more details in Malsiner-Walli et al. (2016). Subsequently, we describe how this approach can be applied to identify cluster-specific characteristics for the sparse hierarchical mixture of mixtures model.

First, we estimate the number of non-empty clusters $\hat{K}_0$ on the upper level of the sparse hierarchical mixture of mixtures model. For this purpose, during MCMC sampling for each iteration m the number of non-empty clusters $K_0^{(m)}$ is determined, i.e. the number of clusters to which observations have been assigned for this particular sweep of the sampler:

$$K_0^{(m)} = K - \sum_{k=1}^{K} I\{N_k^{(m)} = 0\}, \tag{B.1}$$

where $N_k^{(m)} = \sum_{i=1}^{N} I\{S_i^{(m)} = k\}$ is the number of observations allocated to cluster k in the upper level of the mixture for iteration m and I denotes the indicator function. Then,

following Nobile (2004) we obtain the posterior distribution of the number K_0 of non-empty clusters $\Pr\{K_0 = h|\mathbf{y}\}, h = 1, \dots, K$, on the upper level from the MCMC output. An estimator of the true number of clusters $\hat{K}_0$ is then given by the value visited most often by the MCMC procedure, i.e. the mode of the (estimated) posterior distribution $\Pr\{K_0 = h|\mathbf{y}\}$.

After having estimated the number of non-empty clusters $\hat{K}_0$, we condition the subsequent analysis on a model with $\hat{K}_0$ clusters by removing all draws generated in iterations where the number of non-empty clusters does not correspond to $\hat{K}_0$. Among the remaining M_0 draws, only the non-empty clusters are relevant. Hence, we remove all cluster-specific draws $\boldsymbol{\theta}_k$ for empty clusters (which have been sampled from the prior). The cluster-specific draws left are samples from $\hat{K}_0$ non-empty clusters and form the basis for clustering the vector-valued functionals of the draws in the point process representation into $\hat{K}_0$ groups.

It should be noted, that using only vector-valued functionals of the unique parameters $\boldsymbol{\theta}_k$ for this clustering procedure has two advantages. First, $\boldsymbol{\theta}_k$ is a fairly high-dimensional parameter of dimension $d = L - 1 + Lr(r + 3)/2$, in particular if r is large, and the vector-valued functional allows to consider a lower dimensional problem (see also Frühwirth-Schnatter, 2006, 2011). In addition, we need to solve the label switching issue only on the upper level of the sparse hierarchical mixture of mixtures model. Thus, we choose vector-valued functionals of the cluster-specific parameters $\boldsymbol{\theta}_k$ that are invariant to label switching on the lower level of the mixture for clustering in the point process representation of the upper level. We found it particularly useful to consider the cluster means on the upper level mixture, defined by $\boldsymbol{\mu}_k^{(m)} = \sum_{l=1}^{L} w_{kl}^{(m)} \boldsymbol{\mu}_{kl}^{(m)}$.

Clustering the cluster means in the point process representation results in a classification sequence $\rho^{(m)}$ for each MCMC iteration m indicating to which class a single cluster-specific draw belongs. For this, any clustering algorithm could be used, e.g., K-means (Hartigan and Wong, 1979) or K-centroids cluster analysis (Leisch, 2006) where the distance between a point and a cluster is determined by the Mahalanobis distance, see Malsiner-Walli et al. (2016, Section 4.2) for more details. Only the classification sequences $\rho^{(m)}$ which correspond to permutations of $(1, \dots, \hat{K}_0)$ are used to relabel the draws. To illustrate this step, consider for instance, that for $\hat{K}_0 = 4$, for iteration m a classification sequence $\rho^{(m)} = (1, 3, 4, 2)$ is

obtained through the clustering procedure. That means that the draw of the first cluster was assigned to class one, the draw of the second cluster was assigned to class three and so on. In this case, the draws of this iteration are assigned to different classes, which allows to relabel these draws. As already observed by Frühwirth-Schnatter (2006), all classification sequences $\rho^{(m)}$, $m = 1, \ldots, M$ obtained in this step are expected to be permutations, if the point process representation of the MCMC draws contains well-separated simulation clusters.

Nevertheless, it might happen that some of the classification sequences $\rho^{(m)}$ are not permutations. E.g., if the classification sequence $\rho^{(m)} = (3, 1, 2, 1)$ is obtained, then draws sampled from two different clusters are assigned to the same class and no unique labels can be assigned. If only a small fraction $M_{0,\rho}$ of non-permutations is present, then the posterior draws corresponding to the non-permutation sequences are removed from the M_0 draws with $\hat{K}_0$ non-empty clusters. For the remaining $M_0(1 - M_{0,\rho})$ draws, a unique labeling is achieved by relabeling the clusters according to the classification sequences $\rho^{(m)}$. If the fraction $M_{0,\rho}$ is high, this indicates that in the point process representation clusters are overlapping. This typically happens if the selected mixture model with $\hat{K}_0$ clusters is overfitting, see Frühwirth-Schnatter (2011).

This post-processing strategy of the MCMC draws obtained using the sampling strategy described in Appendix A can be summarized as follows:

1. For each iteration $m = 1, \ldots, M$ of the MCMC run, determine the number of non-empty clusters $K_0^{(m)}$ according to (B.1).

2. Estimate the number of non-empty clusters by $\hat{K}_0 = mode(K_0^{(m)})$ as the value of the number of non-empty clusters occurring most often during MCMC sampling.

3. Consider only the subsequence of all MCMC iterations of length M_0 where the number of non-empty clusters $K_0^{(m)}$ is exactly equal to $\hat{K}_0$. For each of the resulting $m = 1, \ldots, M_0$ draws, relabel the posteriors draws $\boldsymbol{\theta}_1^{(m)}, \ldots, \boldsymbol{\theta}_K^{(m)}$, the weight distribution $\eta_1^{(m)}, \ldots, \eta_K^{(m)}$, as well as the upper level classifications $S_1^{(m)}, \ldots, S_N^{(m)}$ such that empty clusters, i.e. clusters with $N_k^{(m)} = 0$, appear last. Remove the empty clusters and keep only the draws $\boldsymbol{\theta}_1^{(m)}, \ldots, \boldsymbol{\theta}_{\hat{K}_0}^{(m)}$ of the $\hat{K}_0$ non-empty clusters.

4. Arrange the $\hat{K}_0$ cluster means $\boldsymbol{\mu}_1^{(m)}, \ldots, \boldsymbol{\mu}_{\hat{K}_0}^{(m)}$ for all M_0 draws in a "data matrix" with $\hat{K}_0 \cdot M_0$ rows and r columns such that the first $\hat{K}_0$ rows correspond to the first draw $\boldsymbol{\mu}_1^{(1)}, \ldots, \boldsymbol{\mu}_{\hat{K}_0}^{(1)}$, the next $\hat{K}_0$ rows correspond to the second draw $\boldsymbol{\mu}_1^{(2)}, \ldots, \boldsymbol{\mu}_{\hat{K}_0}^{(2)}$, and so on. The columns correspond to the different dimensions of $\boldsymbol{\mu}$. Cluster all $\hat{K}_0 \cdot M_0$ draws into $\hat{K}_0$ clusters using either K-means (Hartigan and Wong, 1979) or K-centroids cluster analysis (Leisch, 2006). Either of these cluster algorithms results in a classification index for each of the $\hat{K}_0 \cdot M_0$ rows of the "data matrix" constructed from the MCMC draws. This classification vector is rearranged in terms of a sequence of classifications $\rho^{(m)}$, $m = 1, \ldots, M_0$, where each $\rho^{(m)} = (\rho_1^{(m)}, \ldots, \rho_{\hat{K}_0}^{(m)})$ is a vector of length $\hat{K}_0$, containing the classifications for each draw $\boldsymbol{\mu}_1^{(m)}, \ldots, \boldsymbol{\mu}_{\hat{K}_0}^{(m)}$ at iteration m. Hence, $\rho_k^{(m)}$ indicates for each single draw $\boldsymbol{\mu}_k^{(m)}$ to which cluster it belongs.

5. For each iteration m, $m = 1, \ldots, M_0$, check whether $\rho^{(m)}$ is a permutation of $(1, \ldots, \hat{K}_0)$. If not, remove the corresponding draws from the MCMC subsample of size M_0. The proportion of classification sequences of M_0 not being a permutation is denoted by $M_{0,\rho}$.

6. For the remaining $M_0(1 - M_{0,\rho})$ draws, a unique labeling is achieved by resorting the entire vectors of draws $\{\boldsymbol{\theta}_1^{(m)}, \ldots, \boldsymbol{\theta}_{\hat{K}_0}^{(m)}\}$ (not only $\boldsymbol{\mu}_1^{(m)}, \ldots, \boldsymbol{\mu}_{\hat{K}_0}^{(m)}$), the weight distribution $\eta_1^{(m)}, \ldots, \eta_{\hat{K}_0}^{(m)}$, as well as relabeling the upper level classifications $S_1^{(m)}, \ldots, S_N^{(m)}$ according to the classification sequence $\rho^{(m)}$.

Based on the relabeled draws cluster-specific inference is possible. For instance, a straightforward way to cluster the data is to assign each observation $\mathbf{y}_i$ to the cluster $\hat{S}_i$ which is visited most often. Alternatively, each observation $\mathbf{y}_i$ may also be clustered based on estimating $t_{ik} = \Pr\{S_i = k|\mathbf{y}_i\}$. An estimate $\hat{t}_{ik}$ of t_{ik} can be obtained for each $k = 1, \ldots, K$, by averaging over $\Pr\{S_i = k|\mathbf{y}_i, \boldsymbol{\theta}_k^{(m)}, \eta_k^{(m)}\}$, given by Equation (A.1) using the relabeled draws. Each observation $\mathbf{y}_i$ is then assigned to that cluster which exhibits the maximum posterior probability, i.e. $\hat{S}_i$ is defined in such a way that $\hat{t}_{i,\hat{S}_i} = \max_k \hat{t}_{ik}$. The closer $\hat{t}_{i,\hat{S}_i}$ is to one, the higher is the segmentation power for observation i. Furthermore, the clustering quality of the estimated model can also be assessed based on estimating the posterior expected entropy. The entropy of a finite mixture model is defined in Celeux and Soromenho

(1996) and also described in Frühwirth-Schnatter (2006, p. 28). Entropy values close to zero indicate that observations can unambiguously be assigned to one cluster, whereas large values indicate that observations have high a posteriori probabilities for not only one, but several clusters.

To illustrate identification through clustering the draws in the point process representation in the present context of a mixture of mixtures model, a sparse hierarchical mixture of mixtures model with $K = 10$ clusters and $L = 4$ subcomponents is fitted to the AIS data set (see Figure E.6 and Section 4). The point process representation of the weighted cluster mean draws $\boldsymbol{\mu}_k^{(m)} = \sum_{l=1}^{L} w_{kl}^{(m)} \boldsymbol{\mu}_{kl}^{(m)}$ of *all* clusters, including empty clusters, is shown in Figure B.1 on the left-hand side. Since a lot of draws are sampled from empty clusters, i.e. from the prior distribution, the plot shows a cloud of overlapping posterior distributions where no cluster structure can be distinguished. However, since during MCMC sampling in almost all iterations only two clusters were non-empty, the estimated number of clusters is $\hat{K}_0 = 2$. Thus all draws generated in iterations where the number of non-empty clusters is different from two and all draws from empty clusters are removed. The point process representation of the remaining cluster-specific draws is shown in the scatter plot in the middle of Figure B.1. Now the draws cluster around two well-separated points, and the two clusters can be easily identified.

To illustrate the subcomponent distributions which are used to approximate the cluster distributions, the point process representation of the subcomponent means is shown in Figure B.1 on the right-hand side for the cluster discernible at the bottom right in Figure B.1 in the middle. The plot clearly indicates that all subcomponent means are shrunken toward the cluster mean as the variation of the subcomponent means is about the same as the variation of the cluster means.

C Simulation studies

For both simulation studies, 10 data sets are generated and a sparse hierarchical mixture of mixtures model is estimated. Prior distributions and hyperparameters are specified as described in Section 2.1 and 2.3. MCMC sampling is run for $M = 4000$ iterations after a burn-in of 4000 draws. For the sampling, the starting classification of the observations is

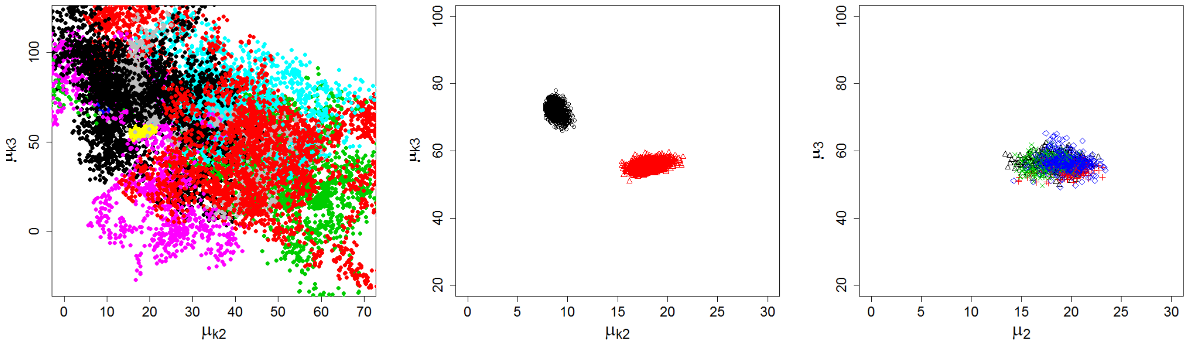

Figure B.1: AIS data set, $K = 10$, $L = 4$, $\phi_B = 0.5$, $\phi_W = 0.1$: Point process representation of the cluster means $\boldsymbol{\mu}_k$ of *all* 10 clusters (left-hand side) and only from those where $\hat{K}_0 = 2$ (middle). Right-hand side: Point process representation of the means of all subcomponents forming the cluster in the bottom right in the plot in the middle.

obtained by first clustering the observations into K groups using K-means clustering and by then allocating the observations within each group to the L subcomponents by using K-means clustering again. The estimated number of clusters is reported in Tables C.1 and C.2, where in parentheses the number of data sets for which this number is estimated is given.

C.1 Simulation setup I

The simulation setup I consists of drawing samples with 800 observations grouped in four clusters. Each cluster is generated by a normal mixture with a different number of subcomponents. The four clusters are generated by sampling from an eight-component normal mixture with component means

$$(\boldsymbol{\mu}_1 \quad \boldsymbol{\mu}_2 \quad \dots \quad \boldsymbol{\mu}_8) = \begin{pmatrix} 6 & 4 & 8 & 22.5 & 20 & 22 & 22 & 6.5 \\ 1.5 & 6 & 6 & 1.5 & 8 & 31 & 31 & 29 \end{pmatrix},$$

variance-covariance matrices

$$\boldsymbol{\Sigma}_1 = \begin{pmatrix} 4.84 & 0 \\ 0 & 2.89 \end{pmatrix}, \qquad \boldsymbol{\Sigma}_2 = \begin{pmatrix} 3.61 & 5.05 \\ 5.05 & 14.44 \end{pmatrix}, \qquad \boldsymbol{\Sigma}_3 = \begin{pmatrix} 3.61 & -5.05 \\ -5.05 & 14.44 \end{pmatrix},$$

$$\boldsymbol{\Sigma}_4 = \begin{pmatrix} 12.25 & 0 \\ 0 & 3.24 \end{pmatrix}, \qquad \boldsymbol{\Sigma}_5 = \begin{pmatrix} 3.24 & 0 \\ 0 & 12.25 \end{pmatrix}, \qquad \boldsymbol{\Sigma}_6 = \begin{pmatrix} 14.44 & 0 \\ 0 & 2.25 \end{pmatrix},$$

$$\boldsymbol{\Sigma}_7 = \begin{pmatrix} 2.25 & 0 \\ 0 & 17.64 \end{pmatrix}, \qquad \boldsymbol{\Sigma}_8 = \begin{pmatrix} 2.25 & 4.2 \\ 4.20 & 16.0 \end{pmatrix},$$

and weight vector $\boldsymbol{\eta} = 1/4(1/3, 1/3, 1/3, 1/2, 1/2, 1/2, 1/2, 1)$.

In Figure C.2 the scatter plot of one data set and the 90% probability contour lines of the generating subcomponent distributions are shown. The first three normal distributions generate the triangle-shaped cluster, the next two the L-shaped cluster, and the last three distributions the cross-shaped and the elliptical cluster. The number of generating distributions for each cluster (clockwise from top left) is 1, 2, 2, and 3. This simulation setup is inspired by Baudry et al. (2010) who use clusters similar to the elliptical and cross-shaped clusters on the top of the scatter plot in Figure C.2. However, our simulation setup is expanded by the two clusters at the bottom which have a triangle and an L shape. Our aim is to recover the four clusters.

If we estimate a sparse finite mixture model (see Malsiner-Walli et al., 2016), which can be seen as a special case of the sparse hierarchical mixture of mixtures model with number of subcomponents $L = 1$, the estimated number of components is seven, as can be seen in the classification results shown in Figure C.2 in the middle plot. This is to be expected, as by specifying a standard normal mixture the number of generating normal distributions is estimated rather than the number of data clusters. In contrast, if a sparse hierarchical mixture of mixtures model with $K = 10$ clusters and $L = 4$ subcomponents is fitted to the data, all but four clusters become empty during MCMC sampling and the four data clusters are captured rather well, as can be seen in the classification plot in Figure C.2 on the right-hand side.

In order to study the effect of changing the specified maximum number of clusters K and subcomponents L on the estimation result, a simulation study consisting of 10 data sets with the simulation setup as explained above and varying numbers of clusters

K \ L	1	3	4	5
4	4(10)	4(10)	4(10)	4(10)
10	7(9)<br>6(1)	4(10)	4(10)	4(10)
15	7(9)<br>8(1)	4(10)	4(9)<br>5(1)	4(10)

Table C.1: Simulation setup I (based on 10 data sets); true number of clusters equal to 4. Results for the estimated number of non-empty clusters $\hat{K}_0$ for various values of K and L. The number of data sets estimating the reported $\hat{K}_0$ is given in parentheses.

$K = 4, 10, 15$ and subcomponents $L = 1, 3, 4, 5$ is performed. For each combination of K and L the estimated number of clusters is reported in Table C.1.

First we study the effect of the number of specified subcomponents L on the estimated number of data clusters. As can be seen in Table C.1, we are able to identify the true number of clusters if the number of subcomponents L forming a cluster is at least three. I.e. by specifying an overfitting mixture with $K = 10$ clusters, for (almost) all data sets superfluous clusters become empty and using the most frequent number of non-empty clusters as an estimate for the true number of data clusters gives good results. If a sparse finite normal mixture is fitted to the data, for almost all data sets 7 normal components are estimated. Regarding the maximum number of clusters K in the overfitting mixture, the estimation results do scarcely change if this number is increased to $K = 15$, as can be seen in the last row of Table C.1. This means that also in a highly overfitting mixture, all superfluous clusters become empty during MCMC sampling.

In Figure C.3, the effect of the number of subcomponents L on the resulting cluster distributions is studied. For the data set shown in Figure C.2, for an increasing number of subcomponents the estimated cluster distributions are plotted using the MAP estimates of the weights, means and covariance matrices of the subcomponents. The estimated cluster distributions look quite similar, regardless of the size of L. This robustness may be due to the smoothing effect of the specified hyperpriors.

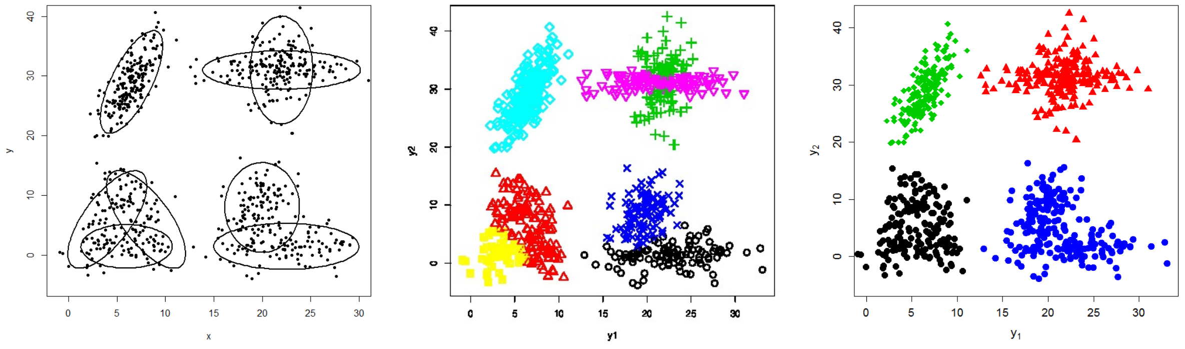

Figure C.2: Simulation setup I. Scatter plot of one data set with the generating component densities shown with 90% probability contour lines (left-hand side), and clustering results by estimating a sparse hierarchical mixture of mixtures model with $K = 10$, $L = 1$ (middle) and $K = 10$, $L = 4$ (right-hand side).

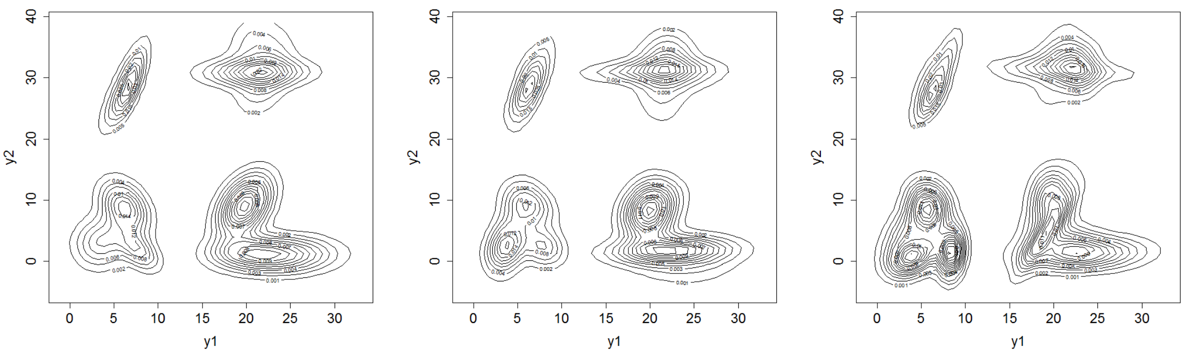

Figure C.3: Simulation setup I, $K = 10$, various values of L. For the data set in Figure C.2, the estimated cluster distributions (MAP estimates of means, weights, and covariance matrices of the subcomponents) are plotted for an increasing number of specified subcomponents $L = 3, 4, 5$ (from left to right).

C.2 Simulation setup II

In Section 2.3 it is suggested to specify the between-cluster variability by $\phi_B = 0.5$ and the between-subcomponent variability by $\phi_W = 0.1$. As can be seen in the previous simulation study in Section C.1 this a priori specification gives promising results if the data clusters are well-separated. However, in contrast to the simulation setup I, in certain applications data clusters might be close or even overlapping. In this case, the clustering result might be sensitive in regard to the specification of ϕ_B and ϕ_W. Therefore, in the following simulation study it is investigated how the specification of ϕ_B and ϕ_W affects the identification of data clusters if they are not well-separated. We want to study how robust the clustering results

are against misspecification of the two proportions.

In order to mimic close data clusters, 10 data sets with 300 observations are generated from a three-component normal mixture, where, however, only two data clusters can be clearly distinguished. In Figure C.4 the scatter plot of one data set is displayed. The 300 observations are sampled from a normal mixture with component means

$$(\boldsymbol{\mu}_1 \quad \boldsymbol{\mu}_2 \quad \boldsymbol{\mu}_3) = \begin{pmatrix} 2 & 4.2 & 7.8 \\ 2 & 4.2 & 7.8 \end{pmatrix},$$

variance-covariance matrices $\boldsymbol{\Sigma}_1 = \boldsymbol{\Sigma}_2 = \boldsymbol{\Sigma}_3 = \mathbf{I}_2$ and equal weights $\boldsymbol{\eta} = (1/3, 1/3, 1/3)$.

For various values of ϕ_B (between 0.1 and 0.9) and ϕ_W (between 0.01 and 0.4) a sparse mixture of mixtures model with $K = 10$ clusters and $L = 4$ subcomponents is fitted and the number of clusters is estimated. For each combination of ϕ_B and ϕ_W the results are reported in Table C.2.

Table C.2 indicates that if ϕ_B increases, also ϕ_W has to increase in order to identify exactly two clusters. This makes sense since by increasing ϕ_B the a priori within-cluster variability becomes smaller yielding tight subcomponent densities. Tight subcomponents in turn require a large proportion ϕ_W of variability explained by the subcomponent means to capture the whole cluster. Thus ϕ_W has to be increased too. However, ϕ_W has to be selected carefully. If ϕ_W is larger than actually needed, some subcomponents are likely to "emigrate" to neighboring clusters. This leads finally to only one cluster being estimated for some data sets. This is basically the case for some of the combinations of ϕ_B and ϕ_W displayed in the upper triangle of the table. In contrast, if ϕ_W is smaller than needed, due to the induced shrinkage of the subcomponent means toward the cluster center, the specified cluster mixture distribution is not able to fit the whole data cluster and two cluster distributions are needed to fit a single data cluster. This can be seen for some of the combinations of ϕ_B and ϕ_W displayed in the lower triangle of the table.

D Description of the data sets

The following data sets are investigated. The Yeast data set (Nakai and Kanehisa, 1991) aims at predicting the cellular localization sites of proteins and can be downloaded from

ϕ_B \ ϕ_W	0.01	0.1	0.2	0.3	0.4
0.1	3(6)<br>2(4)	2(10)	2(5)<br>1(5)	1(8)<br>2(2)	1(8)<br>2(2)
0.3	3(6)<br>2(4)	2(10)	2(8)<br>1(2)	2(6)<br>1(4)	1(7)<br>2(3)
0.5	3(5)<br>2(5)	2(10)	2(10)	2(9)<br>1(1)	2(7)<br>1(3)
0.7	3(7)<br>2(3)	2(7)<br>3(3)	2(10)	2(10)	2(10)
0.9	3(6)<br>4(4)	3(7)<br>2(3)	3(5)<br>2(5)	2(8)<br>3(2)	2(10)

Table C.2: Simulation setup II (based on 10 data sets); true number of clusters equal to 2, $K = 10$, $L = 4$. Results for the estimated number of non-empty clusters $\hat{K}_0$ for various amounts of ϕ_B and ϕ_W. The number of data sets estimating the reported $\hat{K}_0$ is given in parentheses.

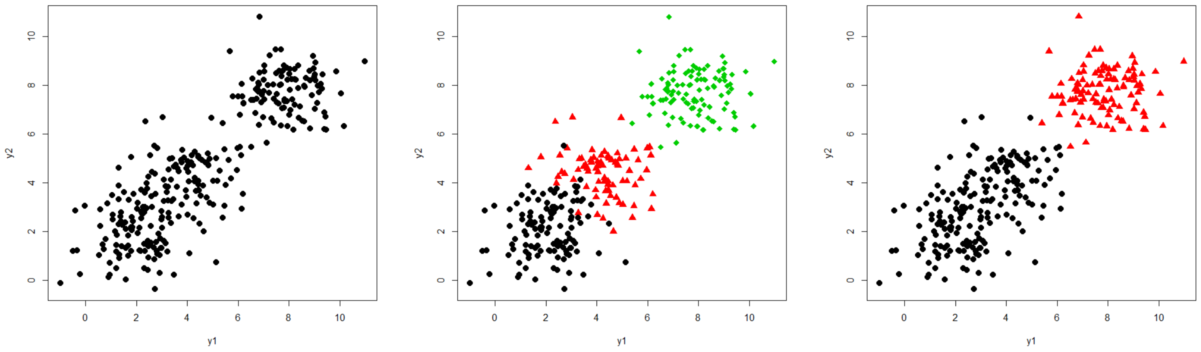

Figure C.4: Simulation setup II. Scatter plot of one data set (left-hand side), classification according to the generating distributions (middle) and to the clusters obtained from a mixture of mixtures with $K = 10$, $L = 4$, $\phi_B = 0.5$ and $\phi_W = 0.1$ (right-hand side).

the UCI machine learning repository (Bache and Lichman, 2013). As in Franczak et al. (2012), we aim at distinguishing between the two localization sites CYT (cytosolic or cytoskeletal) and ME3 (membrane protein, no N-terminal signal) by considering a subset of three variables, namely McGeoch's method for signal sequence (mcg), the score of the ALOM membrane spanning region prediction program (alm) and the score of discriminant analysis of the amino acid content of vacuolar and extracellular proteins (vac).

The Flea beetles data set (Lubischew, 1962) considers 6 physical measurements of 74

male flea beetles belonging to three different species. It is available in the R package **DPpackage** (Jara et al., 2011).

The Australian Institute of Sport (AIS) data set (Cook and Weisberg, 1994) consists of 11 physical measurements on 202 athletes (100 female and 102 male). As in Lee and McLachlan (2013a), we only consider three variables, namely body mass index (BMI), lean body mass (LBM) and the percentage of body fat (Bfat). The data set is contained in the R package **locfit** (Loader, 2013).

The Breast Cancer Wisconsin (Diagnostic) data set (Mangasarian et al., 1995) describes characteristics of the cell nuclei present in images. The clustering aim is to distinguish between benign and malignant tumors. It can be downloaded from the UCI machine learning repository. Following Fraley and Raftery (2002) and Viroli (2010) we use a subset of three attributes: extreme area, extreme smoothness, and mean texture. Additionally, we scaled the data.

The artificial flower data set reported by Yerebakan et al. (2014) can be downloaded from `https://github.com/halidziya/I2GMM`. It consists of 17000 two-dimensional observations representing a flower shape. The data set is generated by seventeen Gaussian densities forming 4 clusters: nine components generate the blossom of the flower, four components the stem and two components each of the two leaves. Note that within each cluster, the generating components have the same orientation. This specification meets the assumption made in the infinite mixture of infinite mixtures model by Yerebakan et al. (2014). We used a subsample of 400 data points for our application, thus leading to the benchmark data sets all being of comparable size. The scatter plot of the sample is given in Figure D.5 on the left-hand side. If we fit a sparse mixture of mixtures model with $K = 10$ clusters and $L = 4$ subcomponents and the usual prior settings as described in Section 2, the four clusters of the flower (petal, stem, and two leaves) can be clearly captured, as can be seen in Figure D.5, where the estimated clustering result and the corresponding cluster distributions are shown.

The flow cytometry data set DLBCL contains intensities of markers stained on a sample of over 8000 cells derived from the lymph nodes of patients diagnosed with Diffuse Large B-cell Lymphoma (DLBCL). The aim of the clustering is to group the individual cell data

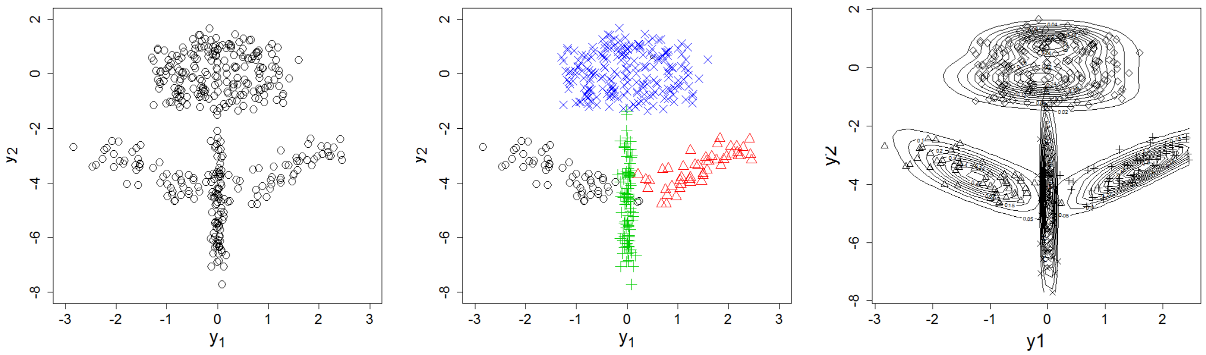

Figure D.5: Flower data set. Boxplot of a sample with 400 data points (left-hand side), the estimated clusters for $K = 10, L = 4$, $\phi_B = 0.5, \phi_W = 0.1, \nu_1 = \nu_2 = 10$ (middle), and the corresponding cluster distributions (right-hand side).

measurements into only a few groups on the basis of similarities in light scattering and fluorescence, see Aghaeepour et al. (2013) for more details. For this data set, class labels of the observations partitioning the data into four classes are available which were obtained by manual partitioning ("gating"). The data set is available in the R package **EMMIXuskew** (Lee and McLachlan, 2013b) as data set `DLBCL` with the corresponding class labels in `true.clusters`.

The flow cytometry data set *GvHDB01case* by Brinkman et al. (2007) consists of 12442 six-dimensional observations which represent a blood sample from a subject who developed Graft versus Host disease (GvHD). GvHD is a severe complication following a blood and marrow transplantation, when donor immune cells in the graft attack the body cells of the recipient. The data were analyzed first by Brinkman et al. (2007). Lo et al. (2008) fitted a Student-t mixture model to this data and estimated 12 clusters using the EM algorithm. In the Bayesian framework, Frühwirth-Schnatter and Pyne (2010) fitted finite mixtures of skew-normal and skew-t distributions and found 12 and 9 clusters. By comparing this sample to a control sample from a patient who had a similar transplantation but did not develop the disease, Brinkman et al. (2007) found a very small cluster of live cells (high "FSC", high "SSC") in the sample with a high expression in the four markers ("CD4+", "CD8β+"', "CD3+", "CD8+"). This cluster was not present in the control sample and seems to be correlated with the development of GvHD.

For the data sets with known class labels, the clustering result of the estimated models is measured by the misclassification rate and the adjusted Rand index (Hubert and Arabie, 1985). To calculate the misclassification rate of the estimated model, the "optimal" matching between the estimated cluster labels and the true known class labels is determined as the one minimizing the misclassification rate over all possible matches for each of the scenarios. The misclassification rate is measured by the number of misclassified observations divided by all observations and should be as small as possible.

The adjusted Rand index (Hubert and Arabie, 1985) is used to assess the similarity between the true and the estimated partition of the data. It is a corrected form of the Rand index (Rand, 1971) which is adjusted for chance agreement. An adjusted Rand index of 1 corresponds to perfect agreement of two partitions whereas an adjusted Rand index of 0 corresponds to results no better than would be expected by randomly drawing two partitions, each with a fixed number of clusters and a fixed number of elements in each cluster.

E Issues with the merging approach

The merging approach, which consists of first fitting a finite mixture of Gaussians to suitably approximate the data distribution and subsequently combines components to clusters, is susceptible to yield poor classifications, since the resulting clusters can only emerge as the union of components that have been identified in the previous step. For illustration, the AIS data (see Appendix D) are clustered using function `clustCombi` (Baudry et al., 2010) from the R package **mclust** (Fraley et al., 2012). The results are shown in Figure E.6. The first step results in a standard Gaussian mixture with three components (left-hand plot), and subsequently *all data* in the smallest component are merged with one of the bigger components to form two clusters (middle plot) which are not satisfactorily separated from each other due to the misspecification of the standard Gaussian mixture in the first step. In contrast, the sparse hierarchical mixture of mixtures approach we develop in the present paper identifies two well-separated clusters on the upper level of the hierarchy (right-hand plot).

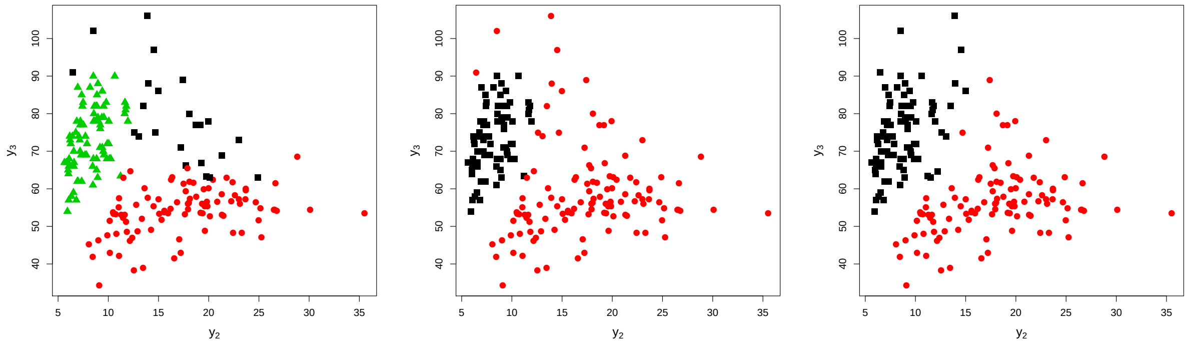

Figure E.6: AIS data set, variables "X.Bfat" and "LBM". Scatter plots of the observations with different estimated classifications based on `Mclust` (left-hand side), `combiClust` (middle), and the sparse hierarchical mixture of mixtures approach developed in this paper ($K = 10, L = 4$) (right-hand side).

F Fitting a mixture of two SAL distributions

Although it is not the purpose of our approach to capture non-dense data clusters, we apply it to the challenging cluster shapes generated by shifted asymmetric Laplace (SAL) distributions, which are introduced by Franczak et al. (2012) in order to capture asymmetric data clusters with outliers. We sampled data from a mixture of two SAL distributions according to Section 4.2 in Franczak et al. (2012). The data set is shown in Figure F.7 on the left-hand side.

If we fit a sparse hierarchical mixture of mixtures model with $K = 10$ clusters and $L = 4$ subcomponents and priors and hyperpriors specified as in Sections 2.1 and 2.3, four clusters are estimated, as can be seen in the middle plot of Figure F.7. Evidently, the standard prior setting, tuned to capture dense homogeneous data clusters, performs badly for this kind of clusters. Thus, in order to take the specific data cluster shapes into account, we adjust the prior specifications accordingly. A data cluster generated by a SAL distribution is not homogeneously dense, it rather consists of a relatively dense kernel on one side of the cluster and a non-dense, light and comet-like tail with possibly extreme observations on the other side. Therefore within a cluster, subcomponents with very different covariance matrices are required in order to fit the whole cluster distribution. Since specification of hyperpriors on λ_{kj} and $\mathbf{C}_{0k}$ has a smoothing and balancing effect on the subcomponent densities, we omit these hyperprior specifications, and choose fixed values for $k = 1, \ldots, K$,

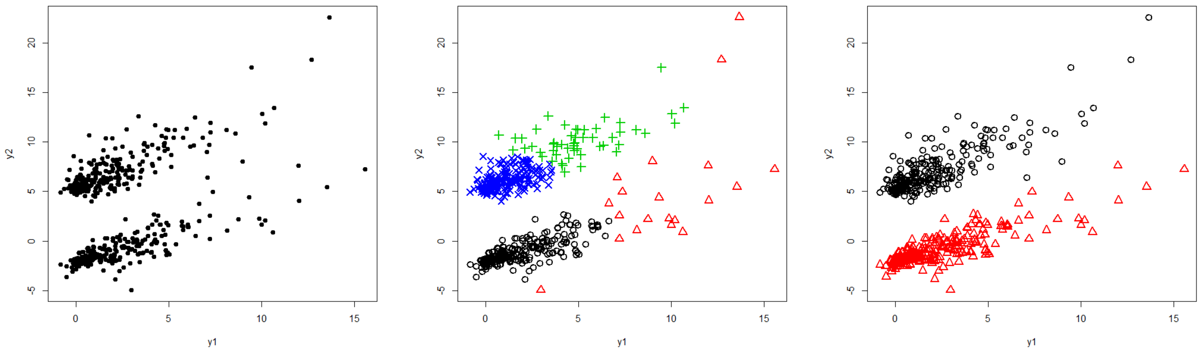

Figure F.7: Samples from a mixture of two *SAL* distributions (left-hand side), the estimated clusters for $K = 10, L = 4$, $\phi_B = 0.5, \phi_W = 0.1, \nu_1 = \nu_2 = 10$ (middle), and for $K = 10, L = 5$, $\phi_B = 0.4, \phi_W = 0.2$, with fixed hyperparameters $\mathbf{C}_{0k} = g_0 \cdot \mathbf{G}_0^{-1}$ and $\lambda_{kj} \equiv 1$ (right-hand side).

i.e. $\mathbf{C}_{0k} = g_0 \cdot \mathbf{G}_0^{-1}$ and $\lambda_{kj} \equiv 1$, $j = 1, \dots, r$.

Additionally, in order to reach also extreme points, we increase both the number of subcomponents to $L = 5$ and the a priori variability explained by the subcomponent means to $\phi_W = 0.2$. At the same time we adjust the proportion of heterogeneity explained by the cluster means by decreasing ϕ_B to 0.4, thus keeping the subcomponent covariance matrices large. If we estimate again a sparse hierarchical mixture of mixtures model with these modified prior settings, the two clusters can be identified, see Figure F.7 on the right-hand side.

References

Aghaeepour, N., G. Finak, H. Hoos, T. R. Mosmann, R. Brinkman, R. Gottardo, R. H. Scheuermann, F. Consortium, D. Consortium, et al. (2013). Critical assessment of automated flow cytometry data analysis techniques. *Nature methods 10*(3), 228–238.

Bache, K. and M. Lichman (2013). UCI machine learning repository. URL `http://archive.ics.uci.edu/ml`.

Baudry, J.-P., A. Raftery, G. Celeux, K. Lo, and R. Gottardo (2010). Combining mixture components for clustering. *Journal of Computational and Graphical Statistics 2*(19), 332–353.

Brinkman, R. R., M. Gasparetto, S.-J. J. Lee, A. J. Ribickas, J. Perkins, W. Janssen, R. Smiley, and C. Smith (2007). High-content flow cytometry and temporal data analysis for defining a cellular signature of graft-versus-host disease. *Biology of Blood and Marrow Transplantation 13*(6), 691–700.

Celeux, G. and G. Soromenho (1996). An entropy criterion for assessing the number of clusters in a mixture model. *Journal of classification 13*(2), 195–212.

Cook, R. D. and S. Weisberg (1994). *An Introduction to Regression Graphics*. Wiley.

Fraley, C. and A. E. Raftery (2002). Model-based clustering, discriminant analysis, and density estimation. *Journal of the American Statistical Association 97*(458), 611–631.

Fraley, C., A. E. Raftery, T. B. Murphy, and L. Scrucca (2012). *mclust Version 4 for R: Normal Mixture Modeling for Model-Based Clustering, Classification, and Density Estimation*. Technical Report 597, Department of Statistics, University of Washington.

Franczak, B. C., R. P. Browne, and P. D. McNicholas (2012). Mixtures of shifted asymmetric Laplace distributions. *eprint arXiv:1207.1727*.

Frühwirth-Schnatter, S. (2006). *Finite Mixture and Markov Switching Models*. New York: Springer.

Frühwirth-Schnatter, S. (2011). Label switching under model uncertainty. In K. Mengerson, C. Robert, and D. Titterington (Eds.), *Mixtures: Estimation and Application*, pp. 213–239. Wiley.

Frühwirth-Schnatter, S. and S. Pyne (2010). Bayesian inference for finite mixtures of univariate and multivariate skew-normal and skew-t distributions. *Biostatistics 11*(2), 317–336.

Hartigan, J. A. and M. A. Wong (1979). Algorithm AS136: A k-means clustering algorithm. *Applied Statistics 28*(1), 100–108.

Hubert, L. and P. Arabie (1985). Comparing partitions. *Journal of Classification 2*(1), 193–218.

Jara, A., T. E. Hanson, F. A. Quintana, P. Müller, and G. L. Rosner (2011). DPpackage: Bayesian semi- and nonparametric modeling in R. *Journal of Statistical Software 40*(5), 1.

Jasra, A., C. C. Holmes, and D. A. Stephens (2005). Markov chain Monte Carlo methods and the label switching problem in Bayesian mixture modelling. *Statistical Science 20*(1), 50–67.

Lee, S. and G. J. McLachlan (2013a). Model-based clustering and classification with non-normal mixture distributions. *Statistical Methods and Applications 22*(4), 427–454.

Lee, S. X. and G. J. McLachlan (2013b). EMMIX-uskew: an R package for fitting mixtures of multivariate skew t-distributions via the EM algorithm. *Journal of Statistical Software 55*(12).

Leisch, F. (2006). A toolbox for K-centroids cluster analysis. *Computational Statistics & Data Analysis 51*(2), 526–544.

Lo, K., R. R. Brinkman, and R. Gottardo (2008). Automated gating of flow cytometry data via robust model-based clustering. *Cytometry Part A 73*(4), 321–332.

Loader, C. (2013). *locfit: Local Regression, Likelihood and Density Estimation.* R package version 1.5-9.1.

Lubischew, A. A. (1962). On the use of discriminant functions in taxonomy. *Biometrics 18*(4), 455–477.

Malsiner-Walli, G., S. Frühwirth-Schnatter, and B. Grün (2016). Model-based clustering based on sparse finite Gaussian mixtures. *Statistics and Computing 26*, 303–324.

Mangasarian, O. L., W. N. Street, and W. H. Wolberg (1995). Breast cancer diagnosis and prognosis via linear programming. *Operations Research 43*(4), 570–577.

Molitor, J., M. Papathomas, M. Jerrett, and S. Richardson (2010). Bayesian profile regression with an application to the National survey of children's health. *Biostatistics 11*, 484–498.

Nakai, K. and M. Kanehisa (1991). Expert system for predicting protein localization sites in gram-negative bacteria. *Proteins: Structure, Function, and Bioinformatics 11*(2), 95–110.

Nobile, A. (2004). On the posterior distribution of the number of components in a finite mixture. *The Annals of Statistics 32*(5), 2044–2073.

Rand, W. M. (1971). Objective criteria for the evaluation of clustering methods. *Journal of the American Statistical Association 66*(336), 846–850.

Viroli, C. (2010). Dimensionally reduced model-based clustering through mixtures of factor mixtures analyzers. *Journal of Classification 27*(3), 363–388.

Yerebakan, H. Z., B. Rajwa, and M. Dundar (2014). The infinite mixture of infinite Gaussian mixtures. In *Advances in Neural Information Processing Systems*, pp. 28–36.
