# Supplementary material for: Identifying Mixtures of Mixtures Using Bayesian Estimation
Source: J Comput Graph Stat. 2017 Apr 24;26(2):285–95. doi: 10.1080/10618600.2016.1200472 (PMC5455957; doi:10.1080/10618600.2016.1200472)
Supplement: Supplementary Materials [file ucgs_a_1200472_sm9526.zip › Code.zip!/Code/Readme.pdf]

README file for "Identifying Mixtures of Mixtures Using Bayesian Estimation"

by Gertraud Malsiner-Walli,
Sylvia Frühwirth-Schnatter, and Bettina Grün

The folder 'Code' contains five 5 files with codes written in R (version 3.0.0) to estimate a sparse hierarchical mixture of mixtures model as defined in Sections 2 and 3 of the paper. The code was tested on the Windows 7 platform. The following R packages are required: **bayesm** 2.2-5, **GIGrvg** 0.1, **MASS** 7.3-26, **MCMCpack** 1.2-4.1, **mclust** 4.0.

- `Simulation_I_II_DataSet.R` is the main file. Data can be generated according to either simulation setup I (Section 4.1) or simulation setup II (Section 4.2). Alternatively the 'Fleabeetles data set' can be used. Then the code in file `Analysis.R` is sourced and the mixture of mixtures model is estimated. The output consists of the initial classification of the observations and the number of observations assigned to the different clusters during MCMC sampling. Finally, the number of estimated clusters, the misclassification rate and the adjusted Rand index (see Section 5 of the paper) are displayed.

- `Analysis.R`: This file contains the code to specify the number of mixture clusters K, subcomponents L, the number of iterations M and burn-in draws *burnin*. Additionally, the values ϕ_B and ϕ_W (see Section 2.3), the prior hyperparameters and initial values for the simulations are defined. The function `MixOfMix_estimation` simulates all parameters from the posterior distribution. The function gives the following posterior draws back:

  - the subcomponent means $\boldsymbol{\mu}_{kl}$ ('Mu'),

- the weighted cluster means $\boldsymbol{\mu}_k$ ('Mu_k'),
- the cluster weights $\boldsymbol{\eta}$ ('eta'),
- the cluster allocations $\mathbf{S}$ of the observations ('S_alt_matrix'),
- the adjustment factors λ_{kr} ('lam_matrixKj'),
- the number of observations assigned to the different clusters ('Nk_matrix_alt'),
- the modes of the the weighted cluster means $\boldsymbol{\mu}_k$ and the modes of the subcomponent means and covariance matrices ('mode_list').

Then the number of non-empty clusters is estimated by $\hat{K}_0$. The function `clust_FS_K0` clusters the weighted cluster mean draws $\boldsymbol{\mu}_k$ into $\hat{K}_0$ clusters, reorders all other draws according to the obtained classifications, removes all non-permutation draws and gives the remaining draws back. The $\hat{K}_0$ clusters with their corresponding samples are now identified. The observations are assigned to the cluster which they were assigned most often to during MCMC sampling. If the true classification is known, the resulting classification error can be computed. The estimated cluster distributions can be plotted.

- `MixOfMix_estimation.R` contains the function of the same name to simulate from the posterior mixture of mixtures distribution according to the MCMC sampling scheme given in Appendix A.

- `clust_FS_K0.R` contains the function of the same name to cluster the draws in the point process representation and delivers the reordered draws.

- `rmvnormMix.R` contains the function of the same name to generate draws from a normal mixture distribution.
